# A Role for E2F Activities in Determining the Fate of Myc-Induced Lymphomagenesis

**DOI:** 10.1371/journal.pgen.1000640

**Published:** 2009-09-11

**Authors:** Rachel E. Rempel, Seiichi Mori, Maura Gasparetto, Michele A. Glozak, Eran R. Andrechek, Steven B. Adler, Nina M. Laakso, Anand S. Lagoo, Robert Storms, Clay Smith, Joseph R. Nevins

**Affiliations:** 1 Duke Institute for Genome Sciences and Policy, Department of Molecular Genetics and Microbiology, Duke University Medical Center, Durham, North Carolina, United States of America; 2 Oncology Research Institute, National University of Singapore, Singapore, Singapore; 3 Terry Fox Laboratory/BC Cancer Research Center, Vancouver, British Columbia, Canada; 4 Department of Interdisciplinary Oncology, Moffitt Cancer Center, Tampa, Florida, United States of America; 5 Department of Pathology, Duke University Medical Center, Durham, North Carolina, United States of America; 6 Department of Medicine, Duke University Medical Center, Durham, North Carolina, United States of America; The University of North Carolina at Chapel Hill, United States of America

## Abstract

The phenotypic heterogeneity that characterizes human cancers reflects the enormous genetic complexity of the oncogenic process. This complexity can also be seen in mouse models where it is frequently observed that in addition to the initiating genetic alteration, the resulting tumor harbors additional, somatically acquired mutations that affect the tumor phenotype. To investigate the role of genetic interactions in the development of tumors, we have made use of the Eμ-*myc* model of pre-B and B cell lymphoma. Since various studies point to a functional interaction between Myc and the Rb/E2F pathway, we have investigated the role of E2F activities in the process of Myc-induced lymphomagenesis. Whereas the absence of E2F1 and E2F3 function has no impact on Myc-mediated tumor development, the absence of E2F2 substantially accelerates the time of tumor onset. Conversely, tumor development is delayed by the absence of E2F4. The enhanced early onset of tumors seen in the absence of E2F2 coincides with an expansion of immature B lineage cells that are likely to be the target for Myc oncogenesis. In contrast, the absence of E2F4 mutes the response of the lineage to Myc and there is no expansion of immature B lineage cells. We also find that distinct types of tumors emerge from the Eμ-*myc* mice, distinguished by different patterns of gene expression, and that the relative proportions of these tumor types are affected by the absence of either E2F2 or E2F4. From these results, we conclude that there are several populations of tumors that arise from the Eμ-*myc* model, reflecting distinct populations of cells that are susceptible to Myc-mediated oncogenesis and that the proportion of these cell populations is affected by the presence or absence of E2F activities.

## Author Summary

The diversity of human cancers reflects the variety of genetic changes that cause tumors to emerge and progress. Even for mice engineered with a specific cancer-causing mutation, the resulting tumors are often divergent, reflecting different additional mutations. We wanted to investigate how activities that work together can collaborate in tumorigenesis. Specifically, we are interested in Myc and the E2F family of proteins, intersecting activities that influence a cell's decision to replicate, rest, or die. We made use of an engineered mouse that develops pre-B and B cell lymphoma initiated by Myc and tested whether the loss of particular E2F family members influences these lymphomas. We found that tumor emergence was accelerated by E2F2 loss and delayed by E2F4 loss. We attributed these results to the finding that the mice lacking E2F2 have a greater proportion than usual of the most susceptible, early-stage B lineage cells and the mice lacking E2F4 have fewer of these cells. Distinct tumor types emerged with their relative proportions influenced by E2F2 and E2F4 status. We conclude that the variety of tumors probably reflect different stages of B lymphoid development that respond to Myc and that E2F proteins can influence the proportions of these different stages.

## Introduction

A hallmark of human cancer is genetic complexity, reflecting the acquisition of multiple mutations and gene rearrangements that give rise to the tumor phenotype. Indeed, recent large-scale DNA sequencing efforts have provided direct evidence for this complexity, revealing large numbers of alterations that characterize various tumor types [1]–[4]. Undoubtedly, this genetic complexity of cancer underlies much of the challenge in developing effective therapeutic strategies. Not only is it likely that combinations of drugs will be necessary to match the complexity and effectively treat these tumors but equally important is the ability to identify subgroups of cancers that represent more homogeneous mechanisms of disease.

An ability to model the complexity that gives rise to the tumor heterogeneity seen in human cancers would clearly enhance the understanding of the oncogenic process but also would enable the development and testing of combination therapeutics that might match this complexity. Mouse models of cancer have generally employed the use of an activated oncogene or the disruption of a tumor suppressor gene to initiate the oncogenic process. Although this represents a defined genetic alteration, it is also true that in most instances this single event is not sufficient to allow for tumor development. This can be seen in the often protracted latency of tumor development as well as the identification of specific additional genetic alterations that appear in these tumors.

An example of a well-studied genetic model for the analysis of pre-B and B cell lymphoma is the Eμ-*myc* transgenic mouse. In the Eμ-*myc* transgenic mouse c-*myc* is constitutively expressed in the B lineage [5],[6]. The resulting polyclonal expansion of pre-B cells is initially limited by increased apoptosis [7]. Additional mutations, many of which inactivate the p53 tumor suppressor pathway [8], then arise. This leads to the emergence of a clonal pre-B or B cell lymphoma by six months of age in mice of a mixed C57Bl/6 and 129 strain background.

Myc has been shown to induce a large number of genes that contribute to cell proliferation. These include the direct transcriptional activation of D cyclin genes, the *cdk4* gene encoding the kinase partner for cyclin D, and the *Cdc25A* gene encoding the phosphatase that removes negative regulatory phosphates from the Cdks. The induction of Cyclin D/cdk4 activity leads directly to the phosphorylation of Rb and thus activation of E2Fs. Numerous studies have demonstrated a central role for the Rb-E2F pathway in the regulation of cellular proliferation. The majority of genes encoding DNA replication and mitotic activities are under the control of E2F proteins. Indeed, recent experiments provide evidence for a role for E2Fs in coordinating transcriptional regulatory events at G1/S and G2/M [9]–[11]. Other work has shown that E2Fs also link this critical proliferative pathway with the p53 response through a capacity to induce the p19ARF/Mdm2 pathway leading to the accumulation of p53 protein [12],[13]. As such, E2Fs provide a mechanism to directly link the control of cell proliferation with the determination of cell fate. In addition to the connection between Myc and E2F in the control of cellular proliferation, Myc expression couples cellular proliferation with the induction of apoptosis under specific growth conditions where survival growth factors are limiting. Myc-induced apoptosis is largely dependent upon p53 signaling and, similar to E2F1, involves the induction of p19^ARF^, inhibition of Mdm2, and elevated p53 [14].

The shared functional properties of the Myc and E2F transcription factors, coupled with the finding that Myc can induce E2F gene expression [15],[16], raise the possibility that Myc function might be mediated, at least in part, through the action of the E2F transcription factors. Indeed, work by the Bernards laboratory revealed that in addition to targeting p27^Kip1^, the mitogenic activity of Myc likely involves regulation of E2Fs [17]. This possibility has been more directly assessed using mouse embryo fibroblasts (MEFs) from embryos deleted for specific E2F genes to evaluate the functional relationship between Myc and various E2F proteins [18]. Experiments using these E2F-deficient MEFs showed that the ability of Myc to induce S phase in the absence of other mitogens is severely impaired in MEFs deleted for *E2f2* or *E2f3*, but not *E2f1* or *E2f4*. In contrast, Myc induced apoptosis in primary serum-deprived MEFs was delayed in cells deleted for *E2f1*, but not affected by *E2f2* or *E2f3* deletion. Thus, at least in cell culture, the induction of specific E2F activities is an essential downstream event in the Myc pathway that controls cell proliferation versus apoptosis, and some of the functions of Myc, such as the induction of p19^ARF^ and p53 could be explained, at least in part, with one pathway leading through E2F activation.

To address the significance of the Myc-E2F connection in a relevant, *in vivo* setting, we have made use of a series of E2F-deficient mouse strains, in combination with the Eμ-*myc* transgenic model of lymphomagenesis (MGI:2448238), to investigate whether deficiencies in E2F1, E2F2, E2F3 or E2F4 (MGI:1857424, 2179111, 2177428 and 1888951) can influence Myc's oncogenic potential. We find that there is a critical role for two E2F activities in affecting the potential for Myc-induced oncogenesis.

## Results

As a prelude to the investigation of a role for E2F activity in Myc-mediated oncogenesis, we analyzed the pattern of tissue-specific expression of the various E2F genes. RNA was prepared from selected tissues of wild type mice and then analyzed by quantitative RT-PCR. *E2f1* and *E2f3* genes were widely expressed whereas expression of the *E2f2* gene was largely restricted to the hematopoietic tissues assayed including bone marrow, spleen, and thymus (Figure S1A). Please note that Text S1 (Supporting Materials and Methods) describes the procedures specific to Figures S1, S2, S3, S4, S5, S6, S7, S8, S9, and S10. This restricted *E2f2* expression pattern at the RNA level was recapitulated at the protein level (Figure S1B). *E2f4*, while expressed widely at the RNA level, was also particularly strongly expressed in hematopoietic tissues at the protein level (Figure S1C) [19]. While we did not measure E2F5 in these assays, it has previously been shown that the expression of this E2F family member is restricted to the differentiating epithelial layers of the skin, intestine and brain [20],[21]. As such, we chose to focus our studies on the *E2f1-4* genes and gene products.

### A role for E2F2 and E2F4 in Myc-induced lymphomagenesis

Male Eμ-*myc* transgenic mice (backcrossed and maintained in the C57BL/6 strain) were bred into the four different E2F-deficient mouse lines. E2F2, E2F3 and E2F4 cohorts were maintained as C57BL/6×129 while the E2F1 cohort was predominantly C57BL/6 in background. In particular, the E2F3 and E2F4 cohorts required maintenance on a mixed, rather than inbred, background because the yield of the *E2f3*-null and *E2f4*-null mice was severely compromised upon inbreeding (data not shown). Sibling mice, wild type, heterozygous or null for a particular E2F gene, and bearing the Eμ-*myc* transgene, were examined weekly for any sign of lymphoma emergence. Each mouse was checked for enlarged lymph nodes, a swollen abdomen, a hunched posture, ruffled fur and/or tachypnea [22]. Upon the appearance of any of these symptoms, the mouse was sacrificed, dissected to identify any lymph node enlargement, and tumor tissue harvested for analysis. For studies assessing the pre-tumor phenotype, mice were sacrificed within three to five weeks after birth and bone marrow and spleen recovered. Such samples were characterized as pre-neoplastic only if lymph node and spleen enlargement was nil or modest at the time of dissection and/or Southern analysis of B lineage cell DNA revealed no specific heavy chain rearrangements indicative of the emergence of tumor clones.

**Figure 1:**
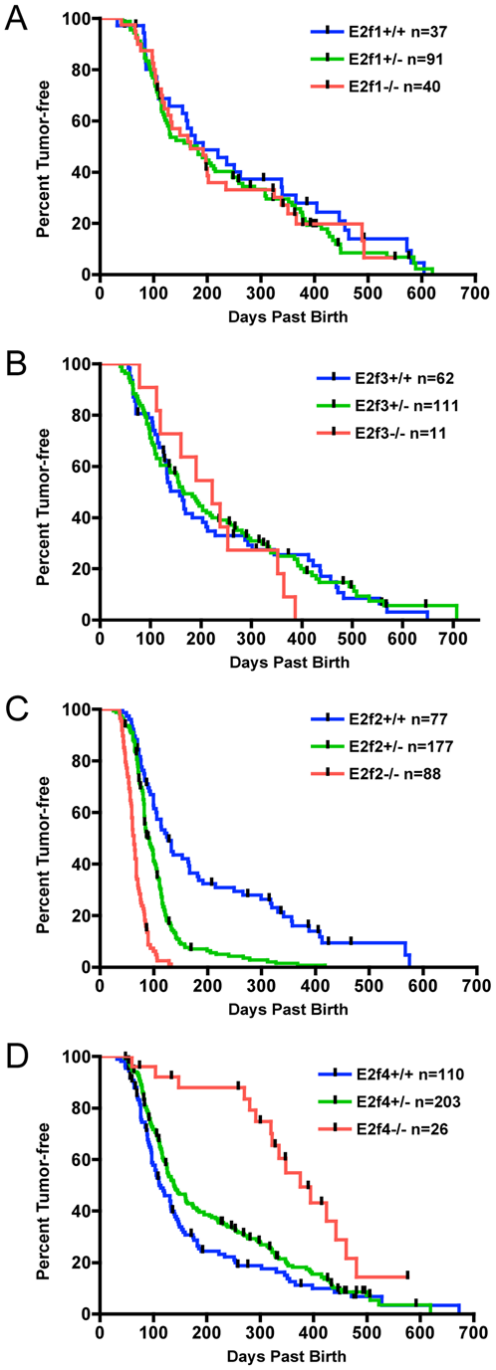
Myc-induced lymphomagenesis is influenced by E2F status. (A) Kaplan-Meier survival analysis showing the percentage of tumor-free Eμ-*myc* mice with the indicated E2F1 genotypes (*E2f1^+/+^*, *E2f1^+/−^*, and *E2f1^−/−^* sibling mice in the E2F1 cohort) plotted against the onset of disease. Onset of disease did not vary with E2F1 status. (B) Same as (A) but for *E2f3^+/+^*, *E2f3^+/−^*, and *E2f3^−/−^* sibling mice in the E2F3 cohort. Onset of disease did not vary with E2F3 status. (C) Same as (A) but for *E2f2^+/+^*, *E2f2^+/−^*, and *E2f2^−/−^* sibling mice in the E2F2 cohort. Disease onset was faster in *E2f2^−/−^* Eμ-*myc* mice (median age 63 days) than in *E2f2^+/+^* Eμ-*myc* mice (median age 126 days), and the onset curves were significantly different (P<0.0001). *E2f2^+/−^* Eμ-*myc* mice exhibited an intermediate onset of disease (median age 92 days), and the onset curve was significantly different from that of the *E2f2^+/+^* Eμ-*myc* mice (P<0.0001) and the *E2f2^−/−^* Eμ-*myc* mice (P<0.0001). (D) Same as (A) but for *E2f4^+/+^*, *E2f4^+/−^*, and *E2f4^−/−^* sibling mice in the E2F4 cohort. Disease onset was slower in *E2f4^−/−^* Eμ-*myc* mice (median age 375 days) than in *E2f4^+/+^* Eμ-*myc* mice (median age 110 days), and the onset curves were significantly different (P<0.0001). Relative to the *E2f4^+/+^* Eμ-*myc* mice, *E2f4^+/−^* Eμ-*myc* mice may have a slightly delayed disease onset (P<0.0465).

Tumor emergence was evaluated in the four E2F cohorts (Figure 1), as well as for our Eμ-*myc* C57BL/6 congenic stock mice (Figure S2A). When the wild type mice in each cohort were compared to the stock transgenic mice, median onsets did diverge (Figure S2B), with earlier onsets associated with greater 129 strain contribution based on breeding history. In spite of this, the overall appearance of each of the wild type curves was similar, with some mice succumbing early and others succumbing late.

As shown in Figure 1A, the loss of E2F1 function did not alter the timing of lymphoma appearance; there was no statistical difference between tumor onset curves when comparing *E2f1^+/+^* (n = 37), *E2f1^+/−^* (n = 91) and *E2f1^−/−^* (n = 40) mice. The failure of E2F1 status to influence lymphomagenesis conflicts with the earlier finding that E2F1 deficiency delays lymphoma development in Eμ-*myc* mice [23]. That study attributed the delay to a defect in p27^Kip1^ degradation and reduced Myc-induced proliferation when E2F1 is reduced or absent. In our assessments the level of p27^Kip1^ protein in splenic B lineage cells did not vary according E2F1 status but rather with progression to disease: p27^Kip1^ was highest in cells isolated from non-transgenic siblings, reduced in healthy Eμ-*myc* transgenics, and lower still in very sick mice and tumors (Figure S3A). In addition, the accelerated proliferation induced by expression of the Eμ-*myc* transgene [24] was unaffected by E2F1 deficiency: splenic B lineage cells isolated from *E2f1* wild type, heterozygous and null Eμ-*myc* transgenic mice all exhibited the same dramatically higher proliferative index when compared to that of cells isolated from non-transgenic siblings (Figure S3B). We note that, analogous to our results, E2F1 deficiency did not alter Myc-induced T cell lymphomagenesis [25]. Given that the timing of Eμ-*myc*-driven tumor development can be influenced by strain background [26] and breeding strategy, we can only surmise that these potential differences or the specific *E2f1*-null allele [27],[28] used in our studies versus that of Baudino and colleagues are sufficient to account for the discrepant effects of E2F1 deficiency.

As shown in Figure 1B, lymphoma onset was also not appreciably influenced by E2F3 status. While the number of *E2f3*-null animals was low in this study, reflecting the low number of *E2f3*-null mice born, there is nevertheless no suggestion that E2F3 loss was protective as several *E2f3*-null mice died before the average age of onset for their wild type siblings.

In contrast to the results seen with the *E2f1* and *E2f3* knockout animals, a deficiency of E2F2 dramatically accelerated the appearance of lymphoma (Figure 1C). The *E2f2^−/−^* mice were prone to early tumor onset with tumors appearing on average 60 days earlier than in their wild-type siblings and there was a significant difference (p<0.0001) between the *E2f2^+/+^* and *E2f2^−/−^* tumor curves. Notably, the *E2f2^+/−^* mice exhibited a median tumor-free span of 92 days and a significantly accelerated course of disease compared to wild-type siblings (p<0.0001). The intermediate phenotype of the *E2f2* heterozygotes suggests a degree of haploinsufficiency. In addition, the *E2f2^+/−^* lymphomas showed no loss of heterozygosity demonstrating that E2F2 does not behave like a classic tumor suppressor in the Eμ-*myc* context (data not shown).

Finally, a deficiency in E2F4 also had a dramatic effect on Myc-induced lymphoma development (Figure 1D). A comparison of *E2f4^+/+^*, *E2f4^+/−^* and *E2f4^−/−^* mice revealed that *E2f4^−/−^* mice remained tumor-free for significantly longer than siblings (p<0.0001) with a median tumor-free span of 375 days past birth. Lymphoma onset may be modestly delayed in *E2f4^+/−^* mice (p = 0.0465).

Taken together, these data would suggest roles for two E2F proteins, both positive and negative acting, in affecting the onset of Myc-mediated lymphomas. A role for these two E2F family members also coincides with the prominent expression of these proteins in hematopoetic tissues (Figure S1).

### Loss of E2F2 or E2F4 function does not alter Myc-mediated proliferation or apoptosis

**Figure 2:**
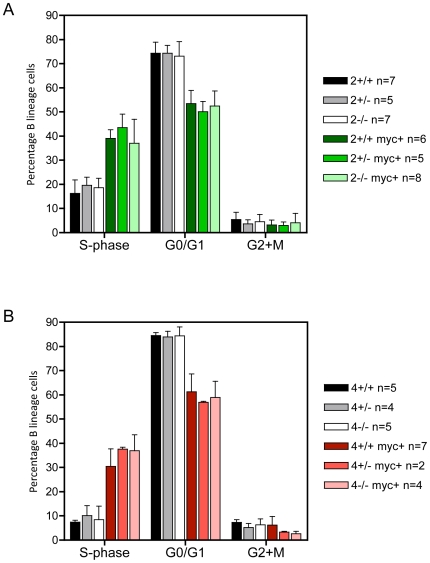
The cell cycle distribution of pretumorous B lineage cells is not altered by E2F2 or E2F4 status. (A) The cell cycle distribution of bone marrow B lineage cells (B220+ CD19+) from pretumorous E2F2 cohort mice. The error bars indicate standard deviation. (B) The cell cycle distribution of bone marrow B lineage cells (B220+ CD19+) from pretumorous E2F4 cohort mice. The error bars indicate standard deviation.

Previous work has shown that in the B cell lineage Myc induces proliferation and apoptosis and retards differentiation [24],[29]. As such, the effects of E2F loss of function on Myc oncogenesis could result from alterations in one or more of these processes. To address the potential for differential effects on proliferation, weanling mice were injected with BrdU, three hours later bone marrow was isolated, and the cell cycle distribution of B lineage cells (B220+ CD19+) determined. As shown in Figure 2A, proliferation of B lineage cells was similar for non-transgenic *E2f2* wild type and null cells. Importantly, the effect of Myc on cell cycle entry of B lineage cells, with increased S-phase cells and reduced G0/G1 cells, was independent of E2F2 status. As shown in Figure 2B, *E2f4* wild type and null B lineage cells were similarly proliferative in non-transgenic mice. As well there was still the expected increase in proliferation associated with bearing the Eμ-*myc* transgene for *E2f4*-null mice (*E2f4^−/−^* compared to *E2f4^−/−^* Eμ-*myc*+: p = 0.0002) (Figure 2B). It appears that the acceleration of cell cycle progression driven by Myc in B lineage cells is not significantly affected by the loss of E2F2 or E2F4 activities.

**Figure 3:**
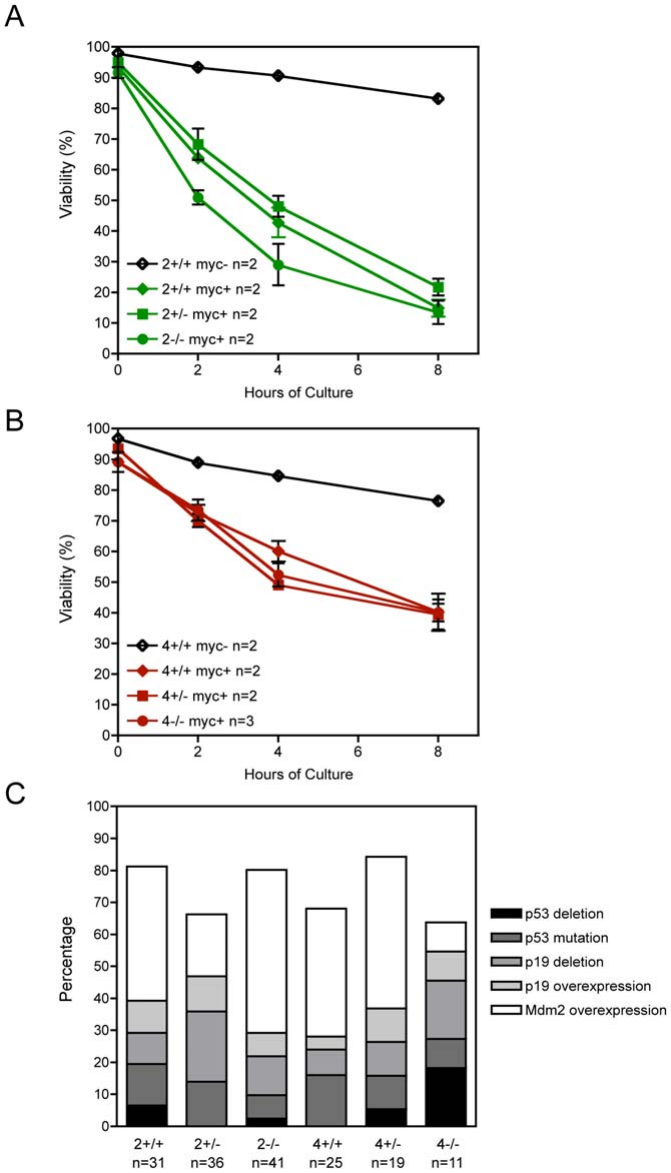
E2F2 and E2F4 do not influence the viability of pretumorous B lineage cells or the incidence of p19Arf-Mdm2-p53 alterations in Myc-induced lymphomas. (A) Representative experiment assessing the viability of B lineage cells (small pre-B and more mature stages) isolated from pretumorous mice of the E2F2 cohort and cultured in the absence of cytokines. Flow cytometry was used to assess viability over time in culture, with B220+ cells negative for both activated caspase 3 and 7-AAD identified as viable. Error bars show standard deviations. (B) Representative experiment assessing the viability of B lineage cells (small pre-B and more mature stages) isolated from pretumorous mice of the E2F4 cohort and cultured in the absence of cytokines. Error bars show standard deviations. (C) Incidence of p53, p19ARF, and Mdm2 alterations in the lymphomas emerging from Eμ-*Myc* transgenic mice of the indicated E2F genotypes. *p53* and *p19ARF* deletions were identified by Southern blotting, and p53, p19ARF, and Mdm2 overexpression by western blotting. As p19ARF overexpression can result from loss of p53 function, tumors were counted as having p19ARF overexpression only when independent of p53 mutation or deletion. Mdm2 overexpression was counted only if exclusive of defects in p53 or p19ARF.

An alteration in the apoptotic potential of Myc could account for the differences in Myc-initiated tumor onset among wild type, *E2f2*-null and *E2f4*-null animals. Possibly in *E2f2^−/−^* mice apoptosis is reduced whereas in *E2f4^−/−^* mice apoptosis is potentiated. Freshly isolated bone marrow B lineage cells (B220+) from *E2f2^−/−^* Eμ-*myc* transgenics and *E2f4^−/−^* Eμ-*myc* transgenics were found to have comparable percentages of activated caspase-3 positive cells as their wild type and heterozygous Eμ-*myc* siblings, around 0.6%, and all the non-transgenic siblings had less than half this percentage of apoptotic cells (data not shown). Since divergent viability may be masked by clearance *in vivo*, the survival of B lineage cells under culture conditions where deregulated Myc induces apoptosis was assessed [30],[31]. The bulk of B lineage cells, excepting progenitors upstream of small pre-B cells, was enriched by negative selection from the bone marrow, spleen and mesenteric lymph node. The resulting population of small pre-B cells, immature B cells and mature B cells from each mouse was cultured for eight hours in medium lacking cytokines. The cultured cells were sampled at two-hour intervals, and B220+ cells assessed for viability based on activated caspase 3 and 7-AAD staining (Figure S4). As expected, the decline in viability was faster for cells from Eμ-*myc* transgenics than from non-transgenics (Figure S4 and Figure 3A and 3B). Notably, when mice in the E2F2 cohort were compared, cells from *E2f2^−/−^* Eμ-*myc* mice lost viability to a similar extent over eight hours as cells from wild type and heterozygous siblings (Figure 3A). Likewise, in an experiment assessing E2F4 cohort mice, cells from *E2f4^−/−^* Eμ-*myc* mice declined in viability similarly to those from their wild type and heterozygous siblings (Figure 3B). Thus, the faster tumor onset for *E2f2*-deficient mice appears not attributable to a general apoptotic deficiency and the slower tumor onset for *E2f4*-null mice unlikely the result of increased apoptosis, at least in the small pre-B cells and more mature stages assessed here that constitute the large majority of B lineage cells.

Myc-mediated tumor emergence is almost invariably associated with a disabling of the ARF-p53 tumor suppressor pathway [8],[14]. Mutations that are indicative of pathway disruption include: *p53* deletion, *ARF* deletion, or overexpression of ARF, Mdm2 or mutant p53. Examples of these disruptions in a sampling of tumors are shown in Figures S5, S6, and S7. Consistent with the earlier studies, tumors from E2F wild type Eμ-*myc* mice showed evidence of disruption of the ARF-Mdm2-p53 pathway (Figure 3C). The largely late-onset lymphomas from *E2f4*-null mice also demonstrated disruptions in this pathway. Importantly, the spectrum and overall incidence of defects in the *E2f2^+/−^* and *E2f2^−/−^* lymphomas were very similar to that shown by the *E2f2^+/+^* lymphomas. In contrast, other modifiers of Myc-induced lymphomagenesis such as Bim and Bax relieve or modify the strong selective pressure for functional inactivation of this pathway [32],[33]. That loss of ARF-p53 function was still associated with development of tumors in the *E2f2^−/−^* mice further supports the evidence that E2F2 deficiency does not compromise Myc-induced apoptosis (Figure 3A).

### Expansion of B lineage progenitor populations in *E2f2^−/−^* mice

**Figure 4:**
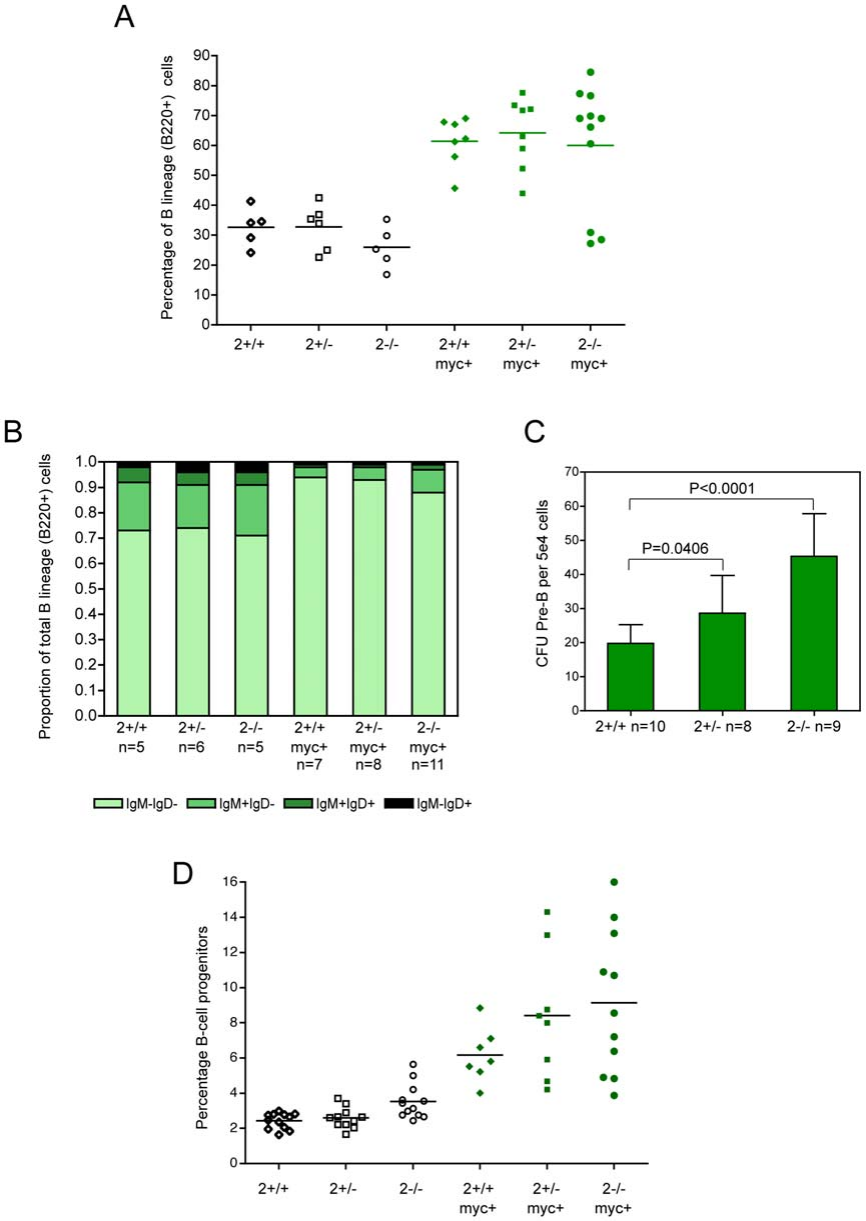
Overall expansion of the B-lineage in response to Myc is not altered by E2F2 status, but *E2f2*-deficient mice have more progenitor B cells. (A) The percentage of B-lineage cells (B220+) within the bone marrow of normal and pretumorous mice with the indicated genotypes. (B) Proportions of bone marrow B lineage cell populations (B220+), from early to more mature stages, across the E2F2 cohort: surface immunoglobulin negative cells, IgM positive IgD negative cells, IgM positive IgD positive cells, and IgM negative IgD positive cells. (C) For clonogenic pre-B cell progenitor assays, an equivalent number of whole bone marrow cells from 8-week old *E2f2^+/+^*, *E2f2^+/−^*, and *E2f2^−/−^* littermates were plated in methylcellulose formulated for pre-B progenitors. After seven days, the number of progenitors (CFU pre-B) per 5×10^4^ input cells was determined by counting the B lineage colonies that had arisen. (D) The proportion of immature B lineage cells (B220+ CD43+) in the bone marrow of normal and pretumorous E2F2 cohort mice of the indicated genotypes.

Given that E2F2 deficiency does not alter the proliferation or apoptosis of pretumorous B lineage cells in response to Myc, we focused on the possibility that there may be a different underlying mechanism driving the accelerated tumor emergence, one involving development of the B lineage and the response to Myc. As noted in one of the earliest descriptions of the Eμ-*myc* model, it is possible that different onsets could reflect different extents of lineage expansion in response to Myc and therefore numbers of vulnerable cells [24]. Overall viable white blood cell number in the bone marrow did not change with E2F2 status while Eμ-*myc* positive mice had modestly higher counts (data not shown). As expected, the B cell lineage expanded as a proportion of the bone marrow in response to the Eμ-*myc* transgene in *E2f2^+/+^* mice (Figure 4A) and the expansion favored less mature over more mature B cells across all three genotypes (Figure 4B). Importantly, there was a similar degree of expansion for the *E2f2^+/−^* and *E2f2^−/−^* mice.

Recent studies have suggested, however, that lymphomagenesis likely initiates in B lineage progenitor cells making the effects of various mutations on progenitor populations particularly relevant [34]. For instance, the Eμ-*myc bcl2^−/−^* mice develop tumors at the same rate as Eμ-*myc bcl2^+/+^* mice despite decreased pre-B, immature B and mature B lymphocytes; significantly, they do have similar numbers of pro-B cells [34]. Similarly, Eμ-*myc/max41* mice develop lymphoma almost as quickly at Eμ-*myc* mice despite a severe deficit in more mature, peripheral B cells [35]. Conversely, the increased progenitor population of early/large pre-B stage cells exhibited by *Phospholipase Cγ2*-deficient mice is associated with accelerated lymphomagenesis [36]. As shown in Figure 4C, CFU pre-B colony assays using bone marrow from non-transgenic E2F2 cohort mice indicate that there were more B lineage progenitors in *E2f2^−/−^* marrow (P<0.0001) and *E2f2^+/−^* marrow (P = 0.0406) than wild type marrow. The proportion of early B lineage cells (B220+ CD43+) identified by flow cytometry was also greater in non-transgenic *E2f2^−/−^* marrow than in non-transgenic *E2f2^+/+^* marrow (P = 0.0008; Figure 4D). The increased proportions of progenitors in *E2f2*-deficient mice extended into pre-tumorous Eμ-*myc* positive mice: there was the trend, although not statistically significant, for more immature B lineage cells as a proportion of the total bone marrow in *E2f2^−/−^* (P = 0.0769) and *E2f2^+/−^* Eμ-*myc* transgenics (P = 0.1393) compared to *E2f2^+/+^* Eμ-*myc* transgenic mice (Figure 4D).

**Figure 5:**
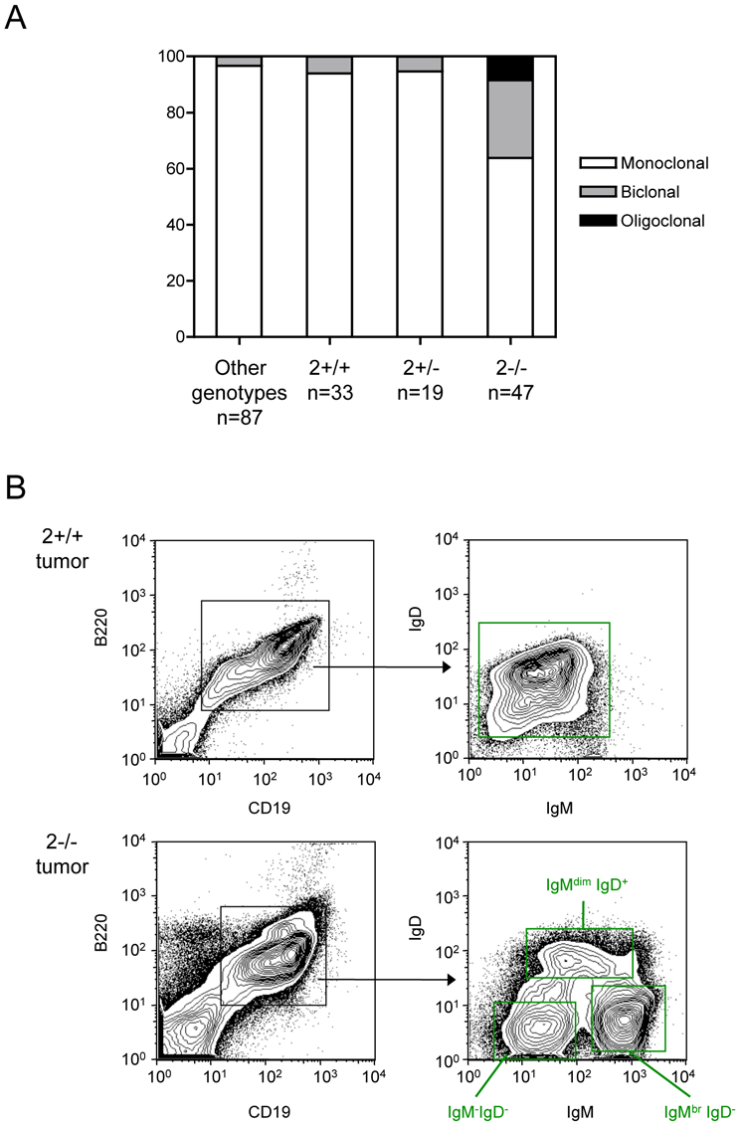
Analysis of E2F2 cohort lymphomas shows that some *E2f2*-null Eμ-*myc* transgenic tumors are polyclonal. (A) The clonality of lymphomas as indicated by the pattern of immunoglobulin heavy chain rearrangements in Southern blotting. Lymphomas were characterized as monoclonal if there were zero, one, or two bands in addition to the residual genomic band, as biclonal if there were three or four bands in addition to the genomic band, and as oligoclonal if there were five or more bands in addition to the genomic band. (B) Flow cytometric analysis of lymphomas. Top panels, an *E2f2^+/+^* Eμ-*myc* transgenic lymphoma and bottom panels, an *E2f2^−/−^* Eμ-*myc* transgenic lymphoma. The tumor cells were stained to identify mature B cell subsets defined by B220 and CD19 and surface expression of IgM and IgD.

Additional analysis revealed that a significant proportion of the *E2f2^−/−^* Eμ-*myc* lymphomas were not monoclonal. Assessment of *Igh* locus rearrangement patterns by Southern analysis indicated that almost 40% of the *E2f2^−/−^* tumors were biclonal or oligoclonal whereas tumors from other genotypes, whether in the E2F2 cohort or in other cohorts, were predominantly monoclonal, in agreement with past studies [5] (Figure 5A and Figure S8A and S8B). This degree of oligoclonality was, however, less extensive than that which occurs with the homozygous Eμ-*myc*/Eμ-*myc* mice [37] and when retrovirally-expressed *myc* was expressed in mice reconstituted with *p53−/−* hematopoietic stem cells [38]. In addition, several tumors were analyzed by flow cytometry for isotypic surface marker expression (Figure 5B). Seven out of ten tumors emerging from *E2f2^−/−^* mice displayed a complex pattern of isotypic surface markers. Such complexity, while not uncommon for Eμ-*myc* lymphomas in general [22], is consistent with multiple clones. Of note, two out of eight tumors from *E2f2^+/−^* mice were similarly complex. In contrast, the majority of tumors from other Eμ-*myc* mice displayed single, homogeneous patterns of surface markers and could be clearly classified as being either pro/pre-B or immature B lymphomas.

Taken together, these findings support the conclusion that in the *E2f2^−/−^* Eμ-*myc* mice there is an increased population of B lineage cells susceptible to lymphomagenesis resulting in the occasional emergence of more than one independent tumor. Using a different Myc transgenic system, Cory and colleagues noted the emergence of mixed T cell tumors in their study and concluded that such mixed tumors originated as separate clones and could be expected with a high rate of tumorigenesis [39]. Also consistent with the hypothesis that the enhancement of early onset tumors in *E2f2^−/−^* mice is the consequence a larger pool of susceptible cells, rather than of differently behaving cells, is the finding that the lymphomas that emerged were very similar to lymphomas that arose early in *E2f2^+/+^* Eμ-*myc* mice. For instance, when mice showing signs of illness were sacrificed the degree of splenomegaly was comparable (Figure S9A) and the histopathology of the lymphomas was similar. The majority of early-onset tumors, either from *E2f2^+/+^* or *E2f2^−/−^* mice, featured high mitotic indices and extensive apoptosis with tingible body macrophages and a starry sky appearance similar to that of human Burkitt lymphoma (data not shown). In addition, B lineage cells (B220+) isolated from *E2f2^+/+^* and *E2f2^−/−^* lymphomas exhibited comparable rates of proliferation and apoptosis (Figure S9B and S9C).

### Reduced populations of B lineage progenitors in *E2f4^−/−^* mice

It has recently been shown that *E2f4*-deficient mice have defects that extend from early hematopoietic progenitor cells, through common lymphoid precursors and into the B and T lineages [40]. Specifically in the B lineage, *E2f4^−/−^* mice exhibit a partial block early in B lineage development prior to immunoglobulin gene rearrangement that results in a deficiency in the most mature pro-B subpopulation and a reduction in more mature B lineage cells [19] (Glozak et al., manuscript in preparation).

**Figure 6:**
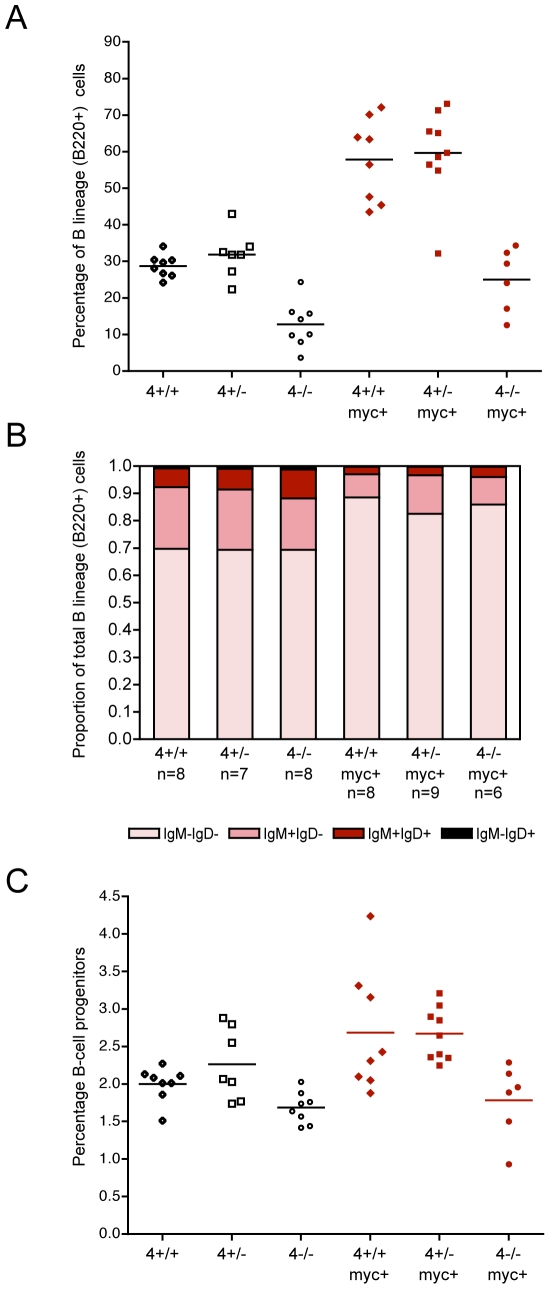
*E2f4* deficiency limits expansion of the B lineage in response to Myc. (A) The percentage of B lineage cells (B220+) within the bone marrow of normal and pretumorous E2F4 cohort mice with the indicated genotypes. (B) Proportions of bone marrow B lineage cell populations (B220+), from early to more mature stages, across the E2F4 cohort: surface immunoglobulin negative cells, IgM positive IgD negative B cells, IgM positive IgD positive B cells, and IgM negative IgD positive B cells. (C) The proportion of immature B lineage cells (B220+ CD43+) in the bone marrow of non-transgenic and pretumorous E2F4 mice of the indicated genotypes.

Total viable white blood cell counts in the bone marrow were modestly higher for Eμ-*myc E2f4^+/+^* and *E2f4^+/−^* mice than for non-transgenic siblings. In the case of *E2f4^−/−^* mice, there was no increase associated with the Eμ-*myc* transgene and Eμ-*myc E2f4^−/−^* mice had about half as many cells as Eμ-*myc* wild type siblings (p = 0.0269). As expected, within the bone marrow, non-transgenic *E2f4^−/−^* mice had a lower proportion of B lineage cells compared to non-transgenic *E2f4^+/+^* mice (P = 0.0133, Figure 6A). Strikingly, the usual expansion of the B lineage in response to the *myc* transgene failed to occur in the *E2f4^−/−^* mice. As a consequence, the proportion of B lineage cells in the bone marrow was significantly less in *E2f4^−/−^* Eμ-*myc* mice than in *E2f4^+/+^* Eμ-*myc* mice (P = 0.0019). Myc did, however, elicit the usual reduction in the relative proportion of mature to less mature B lineage cells in the *E2f4*-deficient mice as in *E2f4* wild type mice (Figure 6B). Motivated by the hypothesis that Eμ-*myc* lymphomas originate in early stage B lineage cells [34], we assessed progenitor populations in the *E2f4^−/−^* mice compared to their siblings. In CFU pre-B colony assays using bone marrow from non-transgenic E2F4 cohort mice, there were significantly fewer progenitors in *E2f4^−/−^* marrow than wild type marrow (data not shown; Glozak et al., manuscript in preparation). Notably, the pre-tumorous *E2f4^−/−^* Eμ-*myc* mice exhibited no expansion of immature B lineage cells (B220+ CD43+) as a proportion of the total bone marrow compared to their non-transgenic *E2f4^−/−^* siblings (Figure 6C). We suggest that the Myc transgene fails to overcome the inefficient developmental progression of the B lineage in *E2f4*-deficient mice, there is a reduced number of susceptible progenitor cells, and consequently delayed tumor emergence.

As indicated by Figure 1D, *E2f4^−/−^* Eμ-*myc* mice displayed a much delayed tumor onset. Possibly because of this delay, of the twenty-six mice assessed, nine died before lymphoma emergence or were still healthy at analysis. The three mice that developed lymphoma early (within 150 days of birth) and one older mouse exhibited the standard Eμ-*myc* lymphoma phenotype, characterized by an enlarged spleen and multiple enlarged lymph nodes. Thirteen mice developed lymphoma very late in life. Three of these mice exhibited lymphoma with modest spleen enlargement and isolated lymph node enlargement, similar the uncommon late onset lymphomas that occasionally develop in Eμ-*myc* mice wild type for *E2F*. The ten remaining *E2f4^−/−^* Eμ-*myc* mice displayed an atypical tumor phenotype that was only rarely noted in *E2F* wild type Eμ-*myc* mice (10 of 17 *E2f4^−/−^* mice compared to only 3 of 79 *E2f4^+/+^* mice). These atypical tumors featured a loose tumor mass of multiple small nodules in the mediastine with little or no associated spleen or peripheral lymph node enlargement. Despite their unusual appearance, the atypical tumors that were tested demonstrated *Igh* gene rearrangement confirming their B lymphoid origin. Overall, eleven *E2f4^−/−^* tumors, including examples of standard, late, and atypical types, were assessed for clonality and all proved to be monoclonal. In summary, along with the general delay in tumor onset there was also a difference in the predominant site of lymphomagenesis and gross morphological appearance of tumors in the *E2f4^−/−^* Eμ-*myc* mice.

### Distinct types of B lineage lymphoma that are influenced by the presence of E2F2 or E2F4

**Figure 7:**
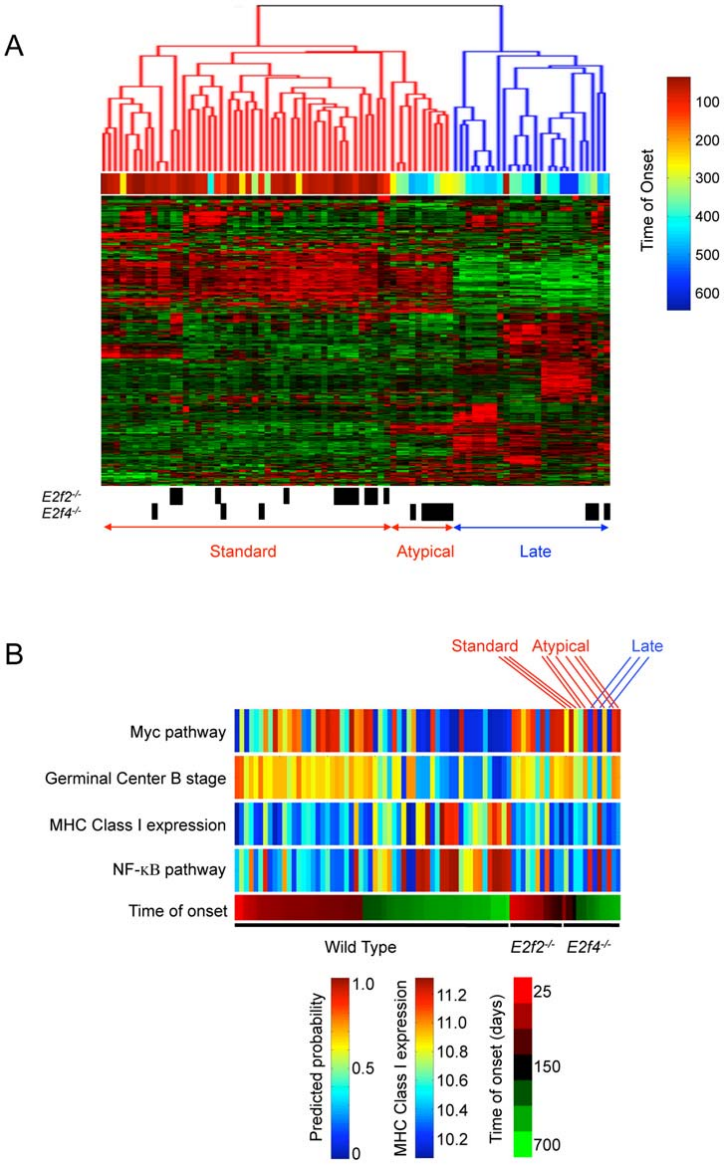
Distinct forms of Eμ-*myc* lymphomas. (A) Unsupervised hierarchical clustering of *E2f2^−/−^* and *E2f4^−/−^* Eμ-*myc* lymphomas amongst *E2F* wild type Eμ-*myc* lymphomas. Dendrogram of tumor subsets and heat map of the expression pattern of 958 genes used for the clustering analysis. Genes with sufficient variation in their expression across wild type (n = 50 plus 8 duplicates), *E2f2^−/−^* (n = 11), and *E2f4^−/−^* (n = 12) tumors were used to identify subgroups of tumors by two-way clustering. The tree classifies the tumors according to similarity of gene expression and identifies two major tumor clusters. The associated time of onset is shown by a color map (red = early onset and blue = late onset). The *E2f2^−/−^* and *E2f4^−/−^* Eμ-*myc* lymphomas and their dissected tumor phenotypes (Standard, Atypical, and Late) are identified. (B) Pathway expression signatures that characterize the distinct forms of Eμ-*myc* lymphomas. *E2F* wild type lymphomas (n = 50), *E2f2^−/−^* lymphomas (n = 11), and *E2f4^−/−^* lymphomas (n = 12) were classified on the basis of Myc pathway activity, the expression of a group of germinal-center B-cell genes, the expression of major-histocompatibility-complex class I genes, and NF-KB pathway activity. The associated dissected tumor phenotypes (Standard, Atypical, or Late) for the *E2f4^−/−^* Eμ-*myc* lymphomas are indicated.

As a further basis for exploring the effects of E2F loss of function on the development of Myc-induced tumors, we have made use of genome-scale gene expression profiles to characterize the tumors arising in the *E2f2*-null and *E2f4*-null Eμ-*myc* mice. Our recent work has identified expression profiles that distinguish different tumor types within the Eμ-*myc* mice including a cluster characterized by generally early onset and pre-B markers as well as three distinct clusters characterized by late onset and different sets of more differentiated B lineage markers [41]. Examples of wild type tumors exhibiting this early and late onset pattern are shown in Figure 7A. Analysis of the tumors from the *E2f2^−/−^* mice indicated that they were relatively homogeneous with respect to their expression profiles and reflected the characteristics of the “early” category of wild type tumors. In contrast, the tumor types from the *E2f4^−/−^* mice were heterogeneous with a distribution across both broad categories of the wild type tumors. The distribution of the *E2f4^−/−^* tumors corresponded with their dissection phenotypes - standard, late and atypical - described above. Three early-onset *E2f4^−/−^* tumors, all with the standard morphological phenotype, clustered with the “early” wild type and *E2f2^−/−^* tumors. Three more *E2f4^−/−^* tumors, all with the late morphological phenotype, clustered alongside a group of wild type tumors that overexpress genes characteristic of plasmacytomas. These particular *E2f4^−/−^* tumors shared marginally decreased *myc* mRNA and low Myc protein compared to other *E2f4^−/−^* tumors (data not shown). The final six *E2f4^−/−^* tumors were all of the atypical phenotype and segregated in the “early” category despite being late onset chronologically. These tumors shared qualities with the “early-standard” tumors in that they featured high levels of *myc* mRNA and Myc protein (data not shown). Notably, these tumors fell at the extreme end of the early cluster and beside a rare group of wild type tumors that had similarly modest spleen enlargement and late chronological onset. In fact, these tumors highlight a significant subgroup within the “early” category that we have designated “early-atypical”. This tumor subgroup was notable for a high incidence of *p53* deletion or mutation (67% of tumors assessed versus 18% of other wild type tumors assessed; by Fisher's exact test P = 0.0061). Among the genes that characterized each tumor cluster, increased expression of number of genes distinguished this subgroup from both “early-standard” and “late” clusters (Figure S10). *Cdkn2a*, the locus that encodes the two tumor suppressors p16(INK4a) and p19(ARF), was preferentially expressed in these tumors. Given that most of these tumors were mutant for *p53*, the increased expression may be a consequence of a role for p53 in negatively regulating the expression of ARF [42]. Other genes that were particularly highly expressed in the “early-atypical” tumors included *Dlk1*, a member of the epidermal growth factor-like family that influences B lineage differentiation [43],[44] and *Fzd6*, a receptor for Wnt signaling and a frizzled family member [45].

To further characterize the distinctions in the Eμ-*myc* tumors, we have made use of signatures of various cell signaling pathways that have previously been shown to distinguish human Burkitt lymphoma (BL) from diffuse large cell B lymphoma (DLBCL) [46]. These include signatures for Myc pathway activity, the expression of a subgroup of germinal-center B-cell genes, the expression of MHC class I genes, and NFκB pathway activity. An analysis of tumors from the *E2f2*-null and *E2f4*-null mice using these pathway signatures is shown in Figure 7B. Consistent with the analysis of whole genome gene expression data in Figure 7A that revealed distinct types of B lymphoma, the analysis using pathway signatures also revealed that the tumors from the *E2f2*-null mice exhibited a pattern similar to the “early” tumors, characterized by high Myc and germinal center signatures, whereas the tumors from *E2f4*-null mice were heterogeneous with respect to these patterns. The atypical *E2f4^−/−^* tumors did not feature the usual “late” characteristics but instead had elevated Myc and germinal center signatures and low MHC Class I and NF-KB signatures. These tumors highlight the existence of certain tumors of late chronological onset, whether *E2F* wild type or *E2f4^−/−^*, that were unusual in their behavior.

## Discussion

A dominant characteristic of the oncogenic process is complexity – the realization that the development of a tumor results from a complex array of genetic alterations that ultimately combine to contribute to the oncogenic phenotype. While transgenic mouse models reduce this complexity by fixing one event, various studies have shown a clear heterogeneity in the tumors that develop reflecting the acquisition of additional alterations. Based on the studies we describe here, we suggest that there are at least two distinct types of B lineage lymphoma that develop in the Eμ-*myc* mice. In mice with wild type E2F function, the predominant tumor arising with an early onset likely reflects the relative abundance of the target cell population. We suggest that the emergence of these tumors reflects a stochastic acquisition of additional mutations. Since this is a probabilistic event, there is then an opportunity for tumors to emerge from other populations of cells with characteristics distinct from the “early-standard” tumors. We propose that the late-onset tumors develop more slowly because the target cell populations are less abundant. It is also possible that these late-onset tumors are genetically more complex, requiring more mutations than the predominant form of tumors.

The work we present here provides clear evidence for an interaction between Myc and two E2Fs as seen by the effect on timing of tumor onset and the different characteristics of the lymphomas that arise in the absence of E2F2 or E2F4. The results suggest distinct roles for the E2F2 and E2F4 proteins in the process of B lineage development that then impacts Myc-mediated oncogenesis. A role for E2F4 in cell differentiation is consistent with past work that documented abnormalities in hematopoietic lineage development as well as the development of the gut and nasal epithelia with loss of E2F4 activity [19], [40], [47]–[49]. This past work revealed a deficiency of various mature hematopoietic cell types and defects in the differentiation of immature cells in the absence of E2F4. Taken together, these observations suggest a critical role for E2F4 activity in controlling the maturation of hematopoietic lineage cells, including the B lymphoid compartment that generates the target cells for Eμ-*myc*-induced lymphomagenesis.

A role for E2F2 appears to be more complex. E2F2 has generally been characterized as one of the E2Fs involved in the activation of transcription of genes essential for cell proliferation. While the other two activating E2Fs (E2F1 and E2F3) appear to be broadly expressed, E2F2 expression is largely restricted to cells of the hematopoietic lineage. Indeed, previous work has pointed to a role for E2F2 in the function and development of various hematopoietic lineages. S-phase progression is impaired in B, erythroid, and myeloid lineages in the absence of E2F1 and E2F2, consistent with a role for these E2F products as positive regulators of cell proliferation [50]. Nevertheless, the possibility of a more complex role for E2F2 in hematopoietic cells emerged from studies demonstrating that the loss of E2F2 appears to enhance the proliferation of T cells following antigenic stimulation, suggesting a negative role for E2F2 in defining a threshold for Ag-stimulated proliferation [51],[52]. Although this latter observation is not consistent with the results we present here regarding proliferative capacity in *E2f2*-null B lineage cells, it certainly is true that with Myc overexpression, the absence of E2F2 function may accelerate tumor onset by increasing the proportion of progenitor B lineage populations.

Opavsky and colleagues have recently used a bitransgenic model of Myc-induced T cell lymphomagenesis to probe the importance of E2F1, E2F2 and E2F3 for Myc activities [25]. Analogous to our findings for B lineage lymphomagenesis, they found that *E2f1* or *E2f3* deficiencies have no effect on T cell lymphoma progression. Most importantly, *E2f2* deficiency accelerates tumor onset in the T cell model as it does for the Eμ-*myc* model. In addition, other aspects of their findings are reminiscent of our results, namely that *E2f2* heterozygotes have an intermediate phenotype, suggesting some haploinsufficiency, and that with *E2f2* deficiency there is an increased incidence of multiple tumor clones. The salient difference is that in their study, *E2f2* deficiency is associated with reduced apoptosis. Specifically, Opavsky and colleagues detected a reduced percentage of annexin-positive T cells in moribund mice that were *E2f2^−/−^* versus *E2f2^+/+^* whereas in our experiments, *E2f2* deficiency was not associated with reduced apoptosis, whether assessing the bulk of B lineage cells from pretumorous mice, or when looking at B220+ cells isolated from tumorous lymph nodes (Figure 3A and Figure S9C).

In marked contrast to the study of Baudino et al. [23], the work described here did not demonstrate that *E2f1* deficiency influenced lymphoma development or p27^Kip1^ regulation. This discrepancy could be the consequence of the different knockout alleles utilized and possible differences in strain background and breeding strategies. But we also note that the wild type *E2f1* Eμ-*myc* mice in the Baudino et al. study [23] exhibit a more precipitous and overall earlier tumor-onset curve than reported in most other studies with C57BL/6 congenic, wild type Eμ-*myc* mice.

### Developmental effects that influence the outcome of oncogenesis

Our results indicate that *E2f2* deficiency enhances the emergence of the “early-standard” form of lymphoma likely because the absence of E2F2 activity expands the population of cells that is the usual target for the oncogenic process in the Eμ-*myc* model. As a result, the population of *E2f2^−/−^* tumors is also more homogeneous with respect to their phenotype, as reflected by the gene expression profiles. In contrast, we propose that the loss of *E2f4* results in the decrease of this population of cells and thus the frequency of appearance of standard morphology early onset tumors. There is not a complete absence of these cells since a few tumors do arise in the absence of E2F4 that cluster with the “early-standard” wild type and *E2f2^−/−^* tumors. But the consequence of this depletion is enrichment for tumors with a late chronological onset, whether to the extreme of the “early” cluster or in the “late” cluster, likely due to an opportunity for these tumors to develop because of the reduced frequency of the “early-standard” variety. Taken together, these results point to a role for E2F activities in determining the population of B lineage cells that contribute to the development of tumors and highlights the interplay between two cell regulatory activities, E2F and Myc, in determining the outcome of the oncogenic process.

## Materials and Methods

### Mouse strains and tumor monitoring

Mice were housed in a Duke University Medical Center Division of Laboratory Animal Resources facility and experiments approved by the Duke University Institutional Animal Care and Use Committee. The generation of the specific lines of *E2F*-deficient mice has been previously described [19],[28],[53]. The original 129 substrain background was 129/SvJae for the E2F1, E2F2 and E2F3 cohorts and 129/OlaHsd for the E2F4 cohort. Based on breeding history, the E2F1 cohort mice used in this study were predominantly C57BL/6 (backcrossed five generations into C57BL/6) while the E2F2, E2F3 and E2F4 cohorts were mixed C57BL/6×129. The four E2F cohorts were maintained separately and breeding involved crossing heterozygous mice to yield wild type, heterozygous and null mice in each generation.

The Eμ-*myc* transgenic mouse line 292-1 [5] extensively backcrossed into C57BL/6 and originally from Dr. Alan Harris (Walter and Eliza Hall Institute, Melbourne, Australia), was kindly provided by Dr. Scott Lowe (Cold Spring Harbor Laboratory). For each E2F cohort, stock Eμ-*myc* positive C57BL/6 congenic males were bred to *E2Fn^+/−^* females and of the progeny only the Eμ-*myc* positive *E2fn^+/−^* males, designated the F1 males, were kept. These F1 males, *E2fn^+/−^ myc^+^*, were then bred to *E2fn^+/−^ myc^−^* females. The Eμ-*myc* positive progeny of this cross, *E2fn^+/+^*, *E2fn^+/−^*, and *E2fn^−/−^* were then compared. Because maternal transmission is associated with reduced latency [34], transmission of the Eμ-*myc* transgene was exclusively paternal in this breeding scheme. Eμ-*myc* negative siblings were also kept as a source of related mice that lacked *myc* transgene effects.

Eμ-*myc* positive mice were monitored weekly to identify any mice with malignant disease. Mice were evaluated for any visible or palpable lumps, a hunched posture, tachypnea, a swollen belly, or ruffled fur and sacrificed promptly upon the appearance of any such symptoms. Lymphomas that emerged were dissected from sacrificed mice, washed in PBS, and frozen in liquid nitrogen or processed for flow cytometric analysis. The frozen tissue provided material for Southern and western analysis.

### Statistical analysis

Tumor onset data refer to the time in days between birth and the first indication of illness. Using GraphPad's Prism program, the time values were plotted to generate Kaplan-Meier survival curves and the curves compared by a logrank test. For comparisons of means and standard deviations, the paired student t-test was performed and statistical significance was determined if the p<0.05.

### Western analysis

To assess alterations in p19ARF, Mdm2 and p53 protein expression, lymphoma samples were dissected from morbid mice and immediately frozen. Samples were then weighed, ground to a powder in liquid nitrogen and resuspended in 60 mM Tris (pH 6.8)/1% SDS at 1 ml per 0.2 g tumor weight, boiled, sonicated, and any remaining debris removed by centrifugation. In parallel, whole cell extracts were made from mouse embryonic fibroblasts infected with the indicated adenoviruses for controls. Protein was quantitated using the BCA Protein Assay Reagent Kit (Pierce). Samples (150 µg) were boiled in sample buffer and subjected to SDS-PAGE on 8.5% polyacrylamide gels for p53 and Mdm2 assessment and 15% gels for ARF assessment. Western analysis was performed as previously described [54]. The blots were probed with antibodies specific for p53 (monoclonal antibody Ab-1 OP03 at 1∶1000, Calbiochem), p19ARF (polyclonal antibody Ab-1 PC435 at 1∶10,000, Calbiochem), and Mdm2 (polyclonal antibody C-18 sc812 at 1∶1000, Santa Cruz Biotechnology). Equal protein loading was verified by staining blots with Ponceau Red (0.2% ponceau red in 3% trichloroacetic acid).

### Southern analysis

Genomic DNA was isolated from lymphomas, normal spleen cells, tail samples and MEFs of specified genotypes. DNA (10 µg) was digested with *Bam*H1 (for the *p53* locus), *Afl*II (for the *p19ARF* locus) or *Eco*RI (for the heavy chain locus). The restricted DNA was separated by agarose gel electrophoresis (0.8% gels), transferred to Hybond N+ membrane, and probed. The *p53* probe was a human cDNA fragment (686 base pair DrdI-StuI fragment extending from exon 4 to exon 10). The *ARF* probe was the exon 1B portion of the *ARF* cDNA (kindly provided by Charles Sherr). The heavy chain locus probe was the heavy chain J3-J4 joining region genomic fragment [37]. On occasion, to verify that weanling mice were essentially tumor-free, genomic DNA isolated from B lineage cells was assessed by Southern analysis for any emergence of clonal heavy chain rearrangements.

### FACS analysis

Mononuclear cells were harvested from the bone marrow of 3-week-old littermates and from lymphomas that arose. Cells were stained with various combinations of antibodies to IgD (11-26c.2a), IgM (R6-60.2), CD19 (1D3), B220 (RA3-6B2), CD43 (S7), BrdU, and active caspase-3. All antibodies and staining reagents were from BD Biosciences. Cell staining procedures were performed either manually or using a Biomek 2000 robotic fluid handler (Beckman Instruments, Schaumburg, IL using a series of mini-programs developed with BioWorks software (Beckman Instruments). FACS analysis was performed on a FACSCalibur device equipped with a 488 nm argon laser and a ∼635 nm red dye laser (Becton Dickinson (BD), San Jose, CA). Data was analyzed using FlowJo Software (TreeStar, Palo Alto, Ca).

### Cell cycle analysis of B lineage cells from normal and pretumorous mice

Three hours before analysis, mice were injected with 100 mg/kg BrdU. Bone marrow mononuclear cells were collected and stained with the B220 and CD19 antibodies to identify B lineage cells, with 7-AAD, and with anti-BrdU antibodies. BrdU Flow Kit reagents and directions were followed (BD/Pharmingen). The proportion of cells that had proceeded through S-phase, or resided in G0/G1 or in G2/M phases was determined.

### Viability assessment in culture of B lineage cells from normal and pretumorous mice

Hematopoietic cells were harvested from the bone marrow, spleen and mesenteric lymph node, combined and enriched for B lineage cells using negative selection (SpinSep Mouse B Cell Enrichment Cocktail, Stem Cell Technologies). The antibodies used to label unwanted cell types for depletion were directed against CD4, CD8, CD11b, CD49b, Gr-1, TER119 and CD43. The approach yielded a subset of B lineage cells from the small pre-B stage through more mature stages. These cells were cultured at 37°C for eight hours in DMEM plus 10%FCS/100 µM L-aspargine/50 µM 2-mercaptoethanol at a concentration of 4×10^6^ cells/ml. At selected time points cells were removed and stained with 7-AAD and B220 antibody, fixed and permeabilized, stained with activated caspase-3 antibody and analyzed by flow cytometry. Viable cells were negative for both 7-AAD and activated caspase-3.

### CFU Pre-B assays

Equivalent numbers of bone marrow cells from non-transgenic 4–6 week old mice were resuspended in Methocult M3630 (Stemcell Technologies) according to manufacturer's specifications to assay for pre-B cell colonies. This media, formulated for the detection and counting of mouse pre-B progenitors in bone marrow, is comprised of methylcellulose in Iscove's MDM supplemented with recombinant IL-7, 2-Mercaptoethanol, L-glutamine, and fetal bovine serum. All samples were assayed in duplicate. After seven days colonies were counted using an inverted microscope. The count was based on the manufacturer's description of expected colony appearance - namely that colonies are composed of at least 30 cells and that individual cells are tiny and irregular to oval in shape.

### DNA microarray analysis

RNA was extracted from lymphoma samples using Qiagen RNeasy Kits (Qiagen). RNA sample integrity was verified by agarose gel electrophoresis or using an Agilent 2100 Bioanalyser. We prepared the targets for DNA microarray analysis and hybridized to Affymetrix Mouse 430 2.0 GeneChip arrays according to the manufacturer's instructions and as previously published. To allow merging of expression array results from samples arrayed independently, some duplicate samples were arrayed to provide reference samples and the expression values standardized using ComBat [55]. The method for cross-platform comparison across different versions of Affymetrix GeneChip arrays was described previously [56].

### Statistical analyses of microarray data

Hierarchical clustering and visualization were performed using Gene Cluster 3.0 (http://bonsai.ims.u-tokyo.ac.jp/~mdehoon/software/cluster/) and Java TreeView (http://jtreeview.sourceforge.net/). Genes and tumors were clustered by average linkage using uncentered correlation as the similarity metric. Analysis of expression data was described previously [56]. In summary, we collected training sets consisting of gene expression values of samples where the pathway activity was known. We created gene expression signatures by choosing the genes whose expression profiles across the training samples most highly correlated with the phenotype. Then, to predict the status of the phenotype on a tumor expression dataset, we fit a Bayesian probit regression model that assigned the probability that a tumor sample exhibited evidence of the phenotype, based on the concordance of its gene expression values with the signature.

The Supporting Materials and Methods are available in Text S1.

## Supporting Information

Figure S1 Tissue-specific expression of E2F family members. (A) Comparison of the relative expression of i) *E2f1* mRNA, ii) *E2f2* mRNA, iii) *E2f3a* and *E2f3b* mRNAs, and iv) *E2f4* mRNA across several tissue types with the expression in bone marrow set to one. Tissues: BM-bone marrow, S-spleen, T-thymus, M-mammary gland, O-ovary, U-uterus, Lu-lung, and Br-brain. (B) Western blot analysis of E2F2 protein in tissues from wild type and matched *E2f2*-null mice. (C) Western blot analysis of E2F4 protein in tissues from wild type and matched *E2f4*-null mice.
(0.96 MB TIF)

Figure S2 Kaplan-Meier survival analysis of Eμ-*myc* C57BL/6 congenic mice compared to the Eμ-*myc E2Fn* wild type mice. (A) Kaplan-Meier survival analysis showing the percentage of tumor-free Eμ-*myc* C57BL/6 congenic mice plotted against the onset of disease. (B) Kaplan-Meier survival analysis comparing the Eμ-*myc* C57BL/6 congenics with the Eμ-*myc E2F* wild type mice from the various cohorts.
(0.22 MB TIF)

Figure S3 Myc-induced degradation of p27^Kip1^ and promotion of proliferation appear independent of E2F1 status. (A) Western blot analysis of p27^Kip1^ in B lineage cells isolated from the spleens of non-transgenic sibling mice and transgenic mice progressing to illness, and from lymphomas. Note that actin levels decrease with disease progression while tubulin levels increase with disease progression. (B) Splenic B lineage cell proliferation in *E2F1* wild type, heterozygous and null mice with and without the Eμ-*myc* transgene. Mice were injected with BrdU, spleens harvested fourteen hours later, and the incorporation of BrdU into DNA in splenic B lineage cells (B220+) assessed by flow cytometry.
(0.78 MB TIF)

Figure S4 Flow cytometric analysis of cultured B lineage cells to assess viability on the basis of staining for activated caspase 3 and 7AAD.‵ B lineage cells (small pre-B and more mature cells) were isolated from the bone marrow, spleen, and mesenteric lymph node and then cultured without cytokines. At indicated times cells were sampled from the cultures and stained for flow cytometry. The boxed region identifies viable B220+ cells. Note that at time zero there were more caspase-positive cells isolated from Eμ-*myc* positive animals than from non-transgenics and that over time caspase-positive cells shifted to becoming 7-AAD positive as well.
(0.74 MB TIF)

Figure S5 Southern analysis of the *p53* locus in normal tail DNA, MEF DNA, and tumor DNA. (A) A 6 kb species and a larger 10 kb pseudogene species were detected upon probing a BamHI digest of mouse genomic DNA using a *p53* human cDNA probe (exons 4 to 10). (B) The mobility of the *p53* locus fragment was altered in DNA isolated from *p53*−/− MEFs. (C) The screening of several Eμ-*myc* lymphoma samples and identification of a single tumor, #14, as having deleted *p53*. Overall, deletion of *p53* was rare in the tumors we assessed, in keeping with the findings of Eischen et al. [8]. The larger 10 kb pseudogene species was unchanged in the *p53*−/− MEFs or any of the tumors and provided a convenient loading control.
(1.57 MB TIF)

Figure S6 Southern analysis of the *ARF* locus in normal tail DNA and tumor DNA. (A) A 7.8 kb fragment representing the *ARF* locus was detected when tail DNA was digested with *Afl*II and probed with *ARF* exon 1B [8]. (B) Assessment of several tumors for *ARF* locus deletion. Tumor #15 exhibited biallelic deletion of *ARF*, while tumor #14, deleted for *p53* (Figure S5), retained *ARF*. (C) The screening of additional Eμ-*myc* tumor samples for *ARF* deletion.
(1.40 MB TIF)

Figure S7 Immunoblot analysis of p53, ARF, and Mdm2 expression in MEFs and Eμ-*myc* tumors. (A) p53 protein was assessed in MEFs infected with either control adenovirus or adenovirus expressing *E2F1*, and in several tumor samples. Forced overexpression of E2F1 induces accumulation of p53 [13], and the same species was evident in one of the tumors. The p53 protein overexpressed in some Eμ-*myc* tumors represents mutant forms that accumulate to very high levels because they fail to induce Mdm2 to trigger their own destruction [8]. (B) ARF protein was assessed in MEFs infected with various recombinant adenoviruses, one of which was Ad-*Arf*, and, in several tumors, some of which overexpress ARF. (C) p53, ARF, and Mdm2 expression assessed in the same set of Eμ-*myc* tumors. While no specific controls were used for Mdm2 protein, assorted species of the expected size and complexity were identified using the antibody referenced by Eischen et al. [8]. In common with earlier studies, p53 overexpression was associated with ARF overexpression, ARF overexpression also occurred independently in additional tumors, and Mdm2 overexpression patterns were complex.
(1.97 MB TIF)

Figure S8 Tumor clonality as determined by *Igh* locus rearrangement patterns. (A) Normal spleen DNA and tumor DNAs were digested with *Eco*RI, agarose gel fractionated, and Southern analysis performed using a heavy chain joining region probe. The arrow indicates the 6.5 kb germline fragment in normal spleen DNA, while in tumor samples additional bands were evident, representing rearranged *Igh* alleles. Most commonly, in addition to the residual germline band, there were two equimolar fragments bearing the heavy chain joining region that resulted from recombination at both *Igh* alleles (see tumor 2 and tumor 4). The single rearranged fragment seen for tumor 1 probably reflects two co-migrating fragments. In the case of tumor 3, the dominance of the germline band suggests that the sample largely comprised normal cells, and indeed the notes describing the dissection suggested that lymphoma had barely initiated. As described in the figure legend for Figure 5A, lymphomas were characterized as monoclonal if there were zero, one, or two bands in addition to any germline band, as biclonal if there were three or four bands in addition to any germline band, and oligoclonal if there were five or more bands in addition to any germline band. (B) As an additional example of this analysis, the *Igh* rearrangement patterns for several *E2f2*+/+ and *E2f2* ^−/−^ Eμ-*myc* tumors are displayed.
(1.25 MB TIF)

Figure S9 The *E2f2*−/− mice exhibit standard Eμ-*myc* lymphoma. (A) Comparison at time of dissection of spleen weights from tumor-bearing *E2f2*+/+ (n = 18), *E2f2*+/− (n = 23), and *E2f2*−/− (n = 16) mice and non-transgenic mice (n = 9). (B) Proliferation of B lineage (B220+) lymphoma cells. Tumorous mice were injected with BrdU and two hours later lymphomas dissected. BrdU incorporation into the DNA of B220+ lymphoma cells was assessed by flow cytometry. (C) Apoptosis of B lineage (B220+) lymphoma cells. Freshly isolated lymphoma cells and normal lymph node cells were isolated and stained with 7-AAD and antibodies to B220 and activated caspase 3. B220+ cells were designated as apoptotic if positive for activated caspase 3 and either negative or positive for 7-AAD.
(0.21 MB TIF)

Figure S10 The expression pattern of genes associated with the Expression levels are displayed with genes as rows and samples as columns and shown by a heatmap in which high expression is indicated by red and low expression by green.
(0.36 MB TIF)

Text S1 Supporting materials and methods.
(0.06 MB DOC)
