# Supplementary figures and images for: A Role for E2F Activities in Determining the Fate of Myc-Induced Lymphomagenesis
Source: PLoS Genet. 2009 Sep 11;5(9):e1000640. doi: 10.1371/journal.pgen.1000640 (PMC2729385; doi:10.1371/journal.pgen.1000640)

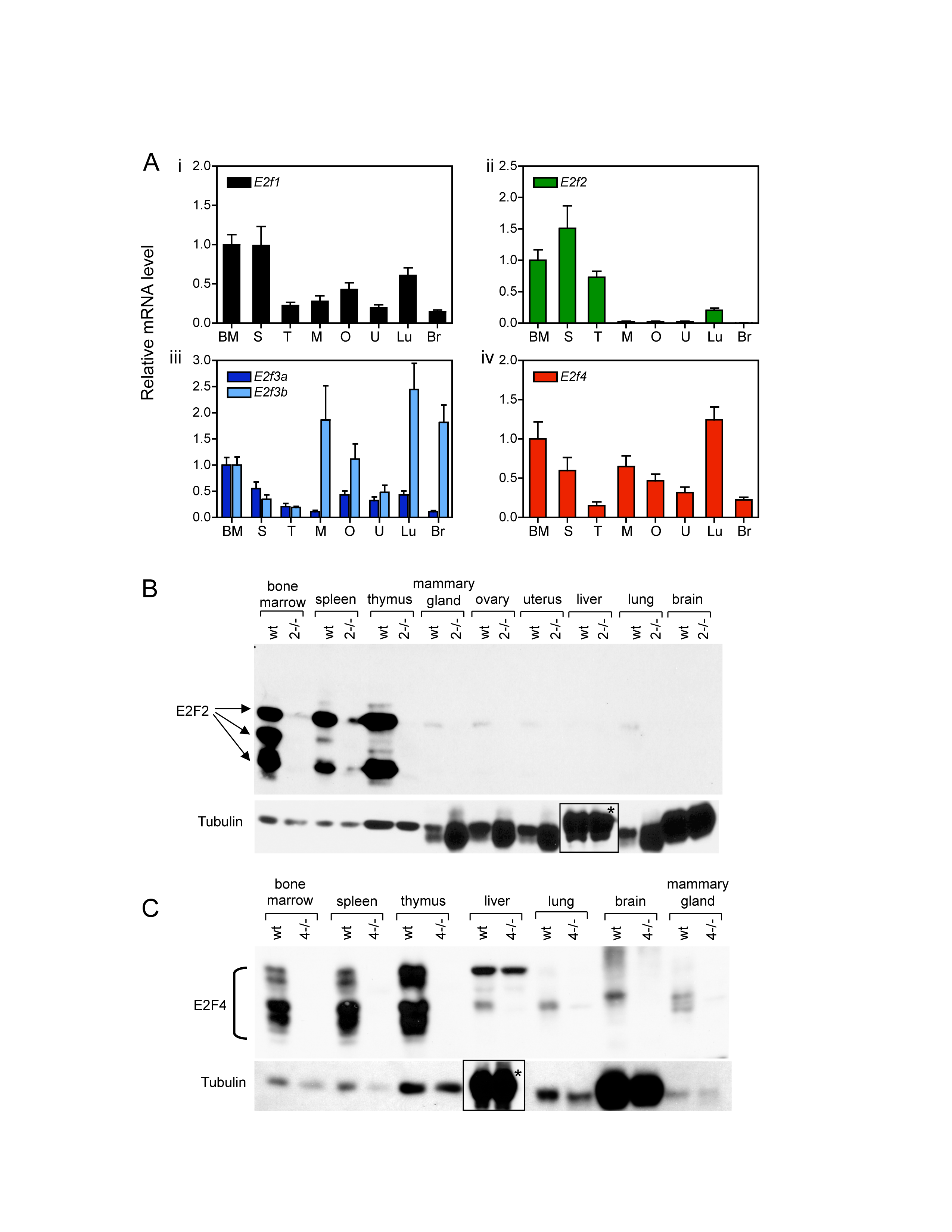

Supplement: Figure S1 — Tissue-specific expression of E2F family members. (A) Comparison of the relative expression of i) E2f1 mRNA, ii) E2f2 mRNA, iii) E2f3a and E2f3b mRNAs, and iv) E2f4 mRNA across several tissue types with the expression in bone marrow set to one. Tissues: BM-bone marrow, S-spleen, T-thymus, M-mammary gland, O-ovary, U-uterus, Lu-lung, and Br-brain. (B) Western blot analysis of E2F2 protein in tissues from wild type and matched E2f2-null mice. (C) Western blot analysis of E2F4 protein in tissues from wild type and matched E2f4-null mice. (0.96 MB TIF) [file pgen.1000640.s001.tif]

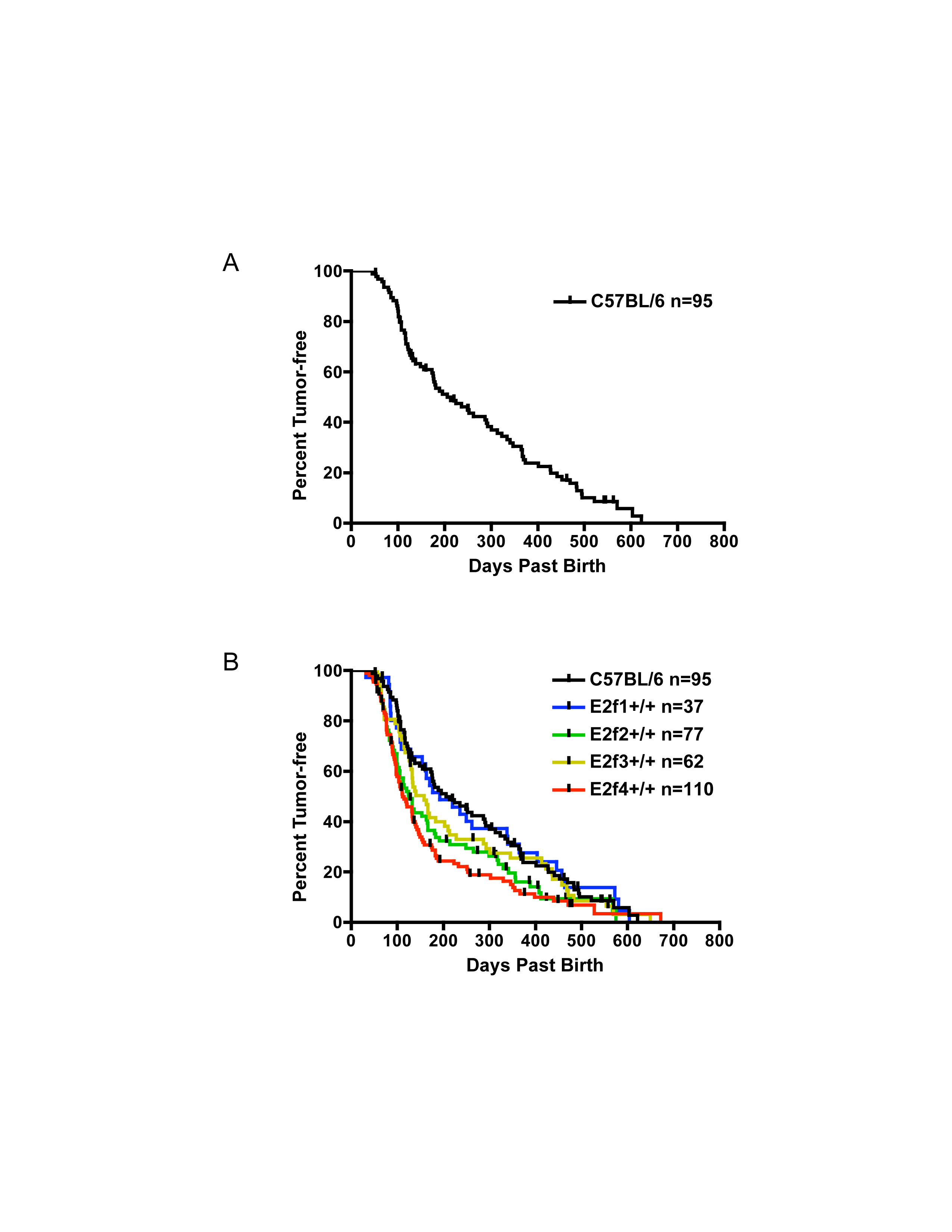

Supplement: Figure S2 — Kaplan-Meier survival analysis of Eμ-myc C57BL/6 congenic mice compared to the Eμ-myc E2Fn wild type mice. (A) Kaplan-Meier survival analysis showing the percentage of tumor-free Eμ-myc C57BL/6 congenic mice plotted against the onset of disease. (B) Kaplan-Meier survival analysis comparing the Eμ-myc C57BL/6 congenics with the Eμ-myc E2F wild type mice from the various cohorts. (0.22 MB TIF) [file pgen.1000640.s002.tif]

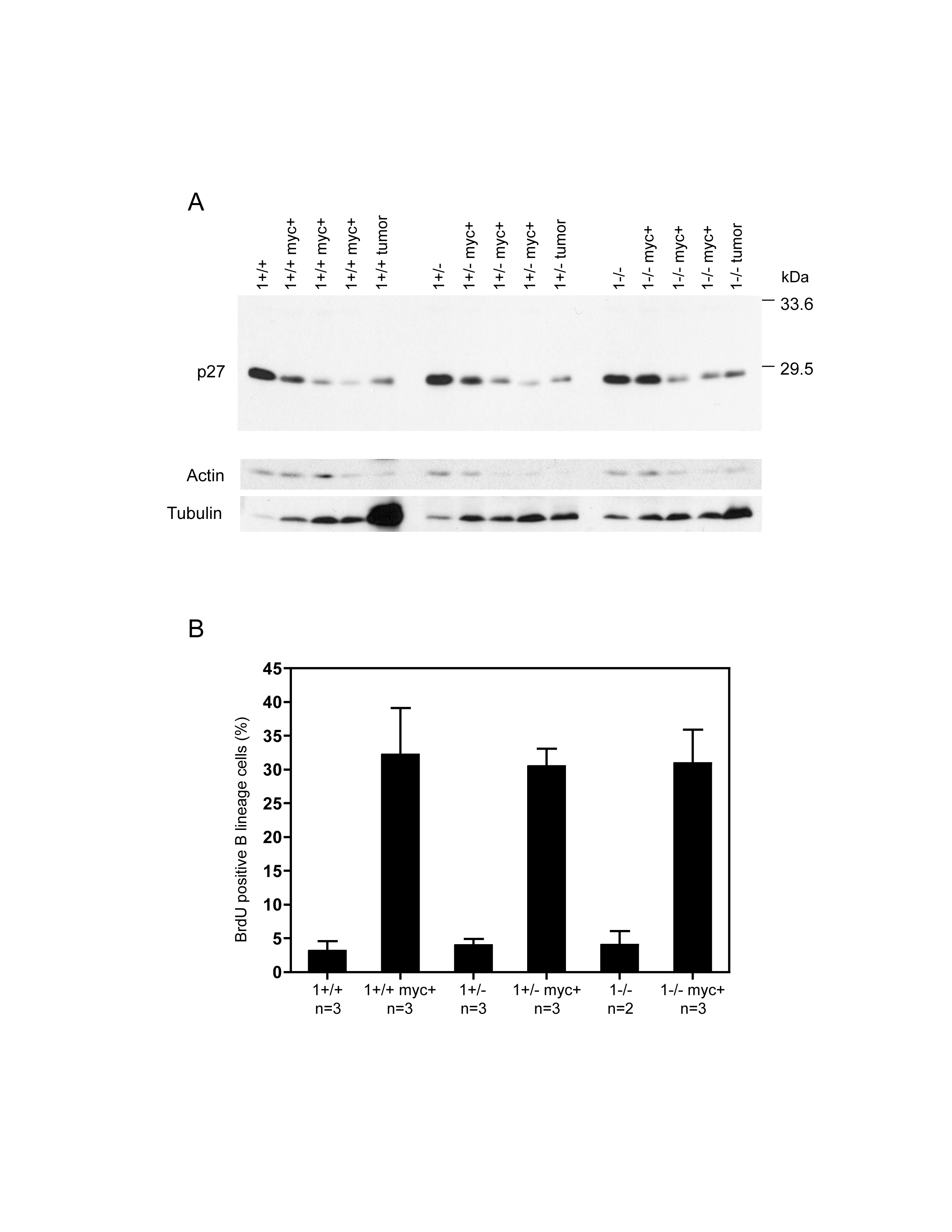

Supplement: Figure S3 — Myc-induced degradation of p27Kip1 and promotion of proliferation appear independent of E2F1 status. (A) Western blot analysis of p27Kip1 in B lineage cells isolated from the spleens of non-transgenic sibling mice and transgenic mice progressing to illness, and from lymphomas. Note that actin levels decrease with disease progression while tubulin levels increase with disease progression. (B) Splenic B lineage cell proliferation in E2F1 wild type, heterozygous and null mice with and without the Eμ-myc transgene. Mice were injected with BrdU, spleens harvested fourteen hours later, and the incorporation of BrdU into DNA in splenic B lineage cells (B220+) assessed by flow cytometry. (0.78 MB TIF) [file pgen.1000640.s003.tif]

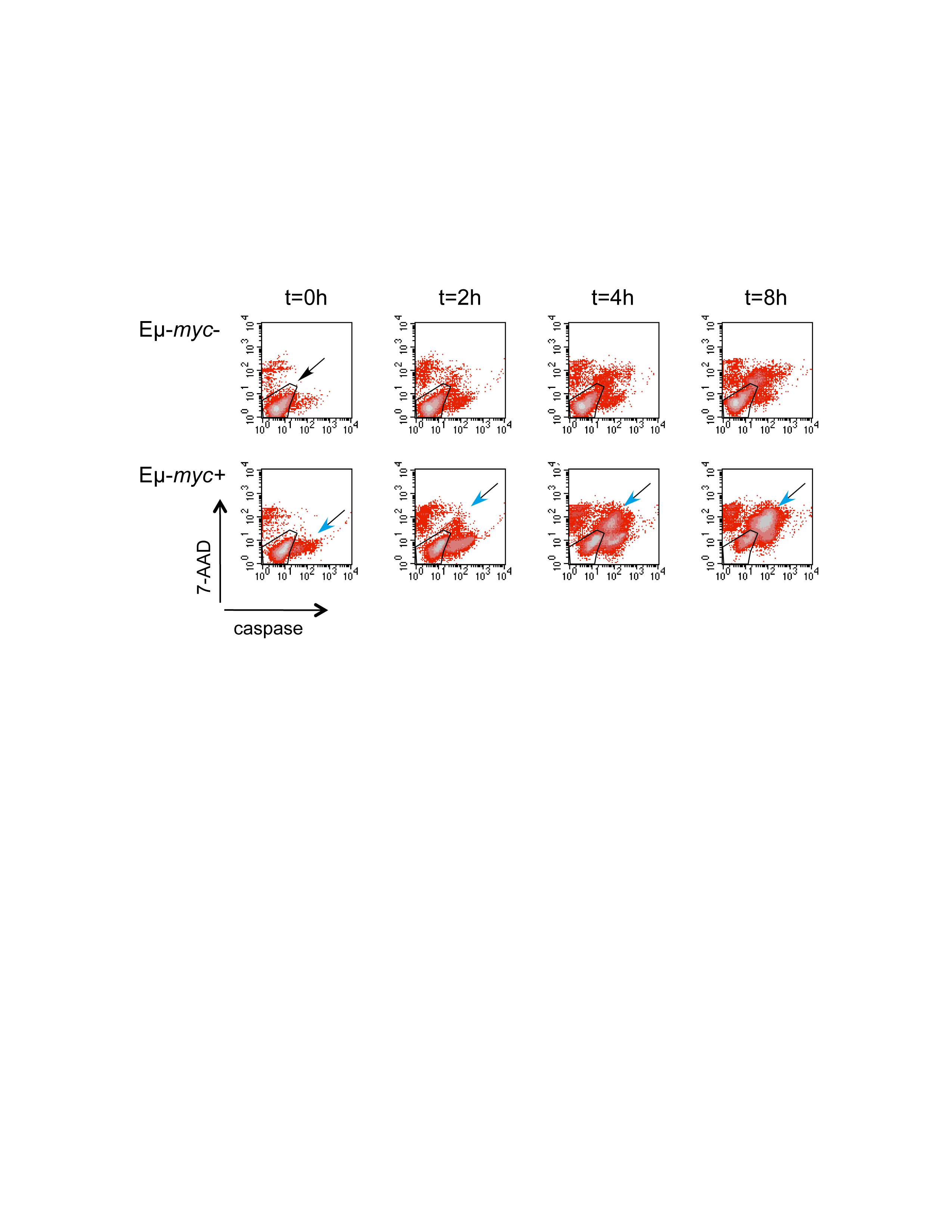

Supplement: Figure S4 — Flow cytometric analysis of cultured B lineage cells to assess viability on the basis of staining for activated caspase 3 and 7AAD.‵ B lineage cells (small pre-B and more mature cells) were isolated from the bone marrow, spleen, and mesenteric lymph node and then cultured without cytokines. At indicated times cells were sampled from the cultures and stained for flow cytometry. The boxed region identifies viable B220+ cells. Note that at time zero there were more caspase-positive cells isolated from Eμ-myc positive animals than from non-transgenics and that over time caspase-positive cells shifted to becoming 7-AAD positive as well. (0.74 MB TIF) [file pgen.1000640.s004.tif]

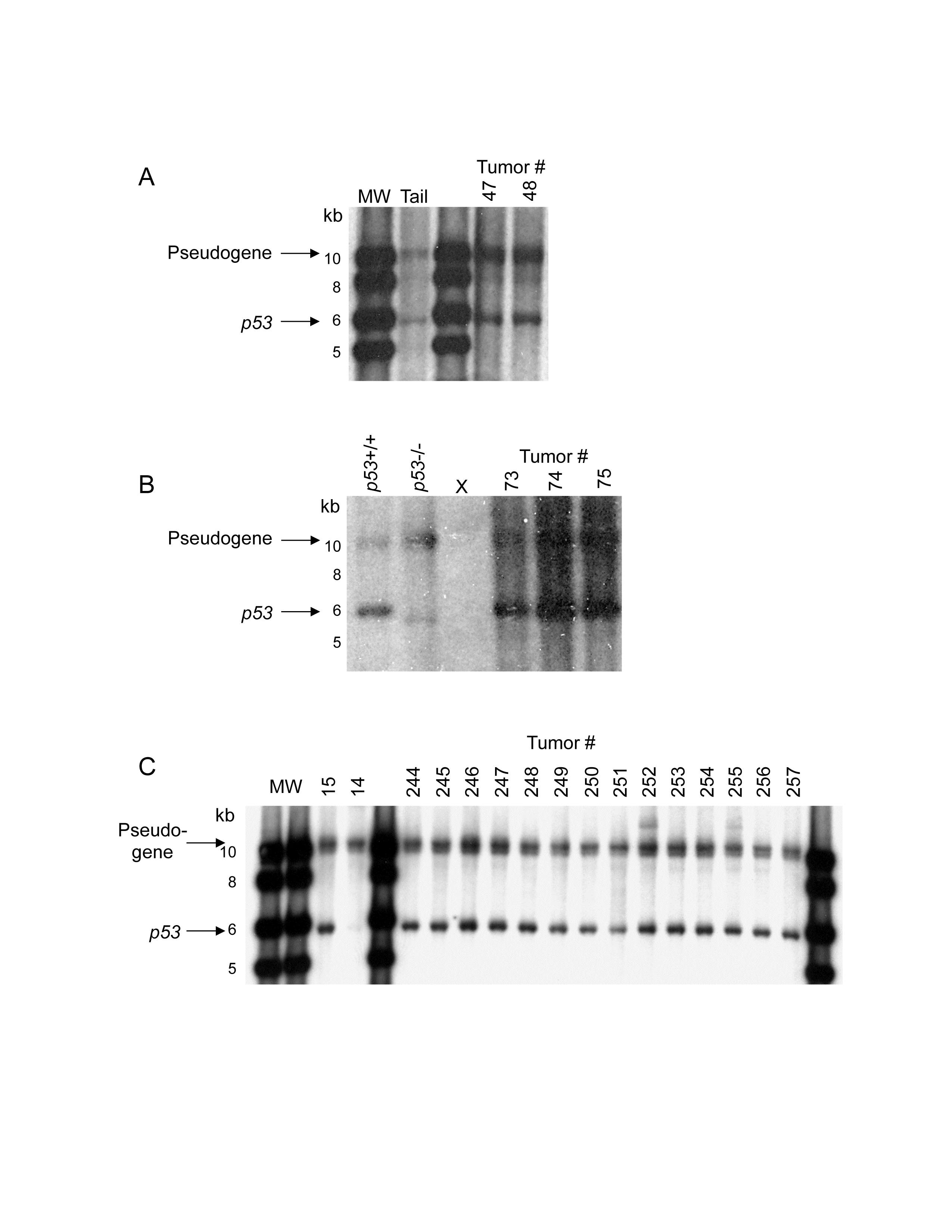

Supplement: Figure S5 — Southern analysis of the p53 locus in normal tail DNA, MEF DNA, and tumor DNA. (A) A 6 kb species and a larger 10 kb pseudogene species were detected upon probing a BamHI digest of mouse genomic DNA using a p53 human cDNA probe (exons 4 to 10). (B) The mobility of the p53 locus fragment was altered in DNA isolated from p53−/− MEFs. (C) The screening of several Eμ-myc lymphoma samples and identification of a single tumor, #14, as having deleted p53. Overall, deletion of p53 was rare in the tumors we assessed, in keeping with the findings of Eischen et al. [8]. The larger 10 kb pseudogene species was unchanged in the p53−/− MEFs or any of the tumors and provided a convenient loading control. (1.57 MB TIF) [file pgen.1000640.s005.tif]

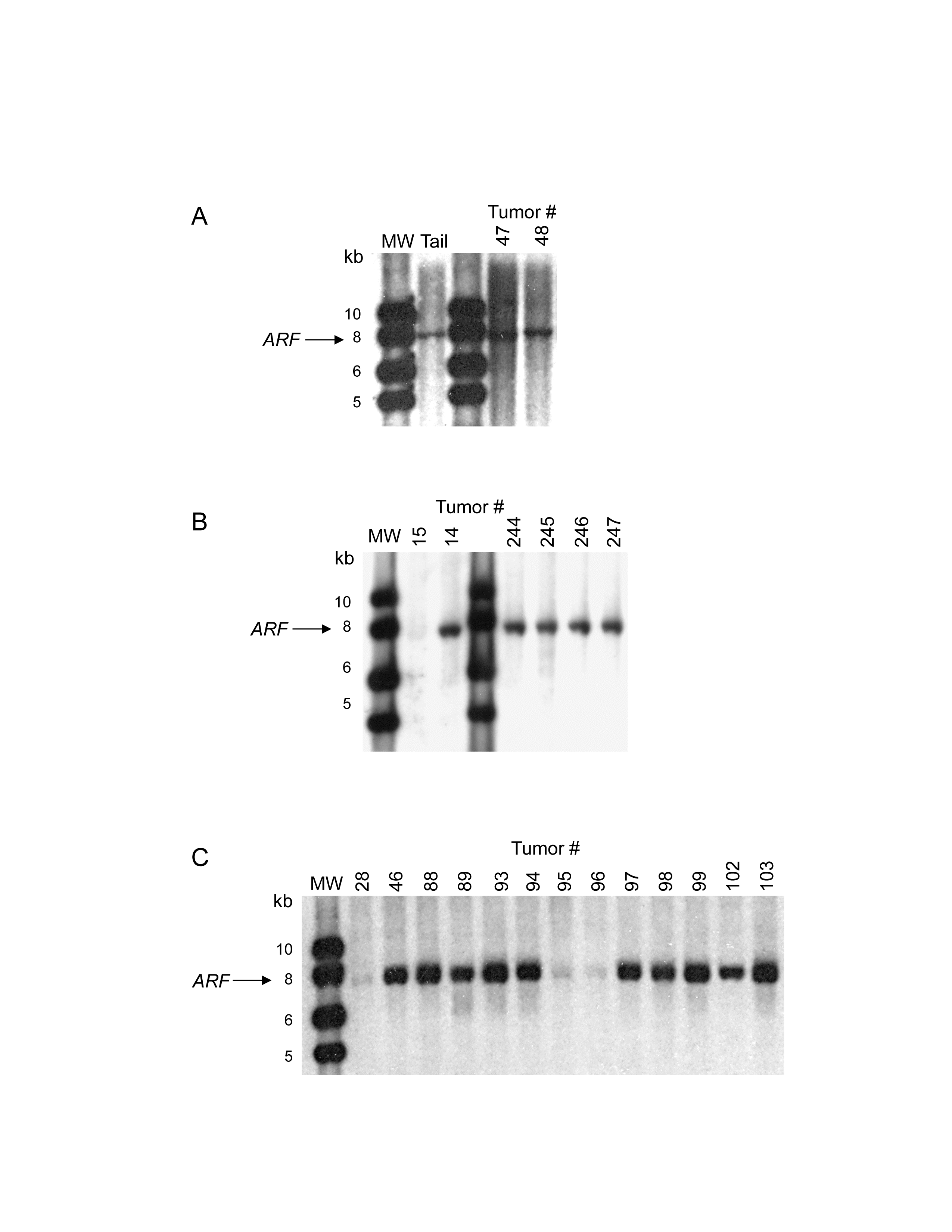

Supplement: Figure S6 — Southern analysis of the ARF locus in normal tail DNA and tumor DNA. (A) A 7.8 kb fragment representing the ARF locus was detected when tail DNA was digested with AflII and probed with ARF exon 1B [8]. (B) Assessment of several tumors for ARF locus deletion. Tumor #15 exhibited biallelic deletion of ARF, while tumor #14, deleted for p53 (Figure S5), retained ARF. (C) The screening of additional Eμ-myc tumor samples for ARF deletion. (1.40 MB TIF) [file pgen.1000640.s006.tif]

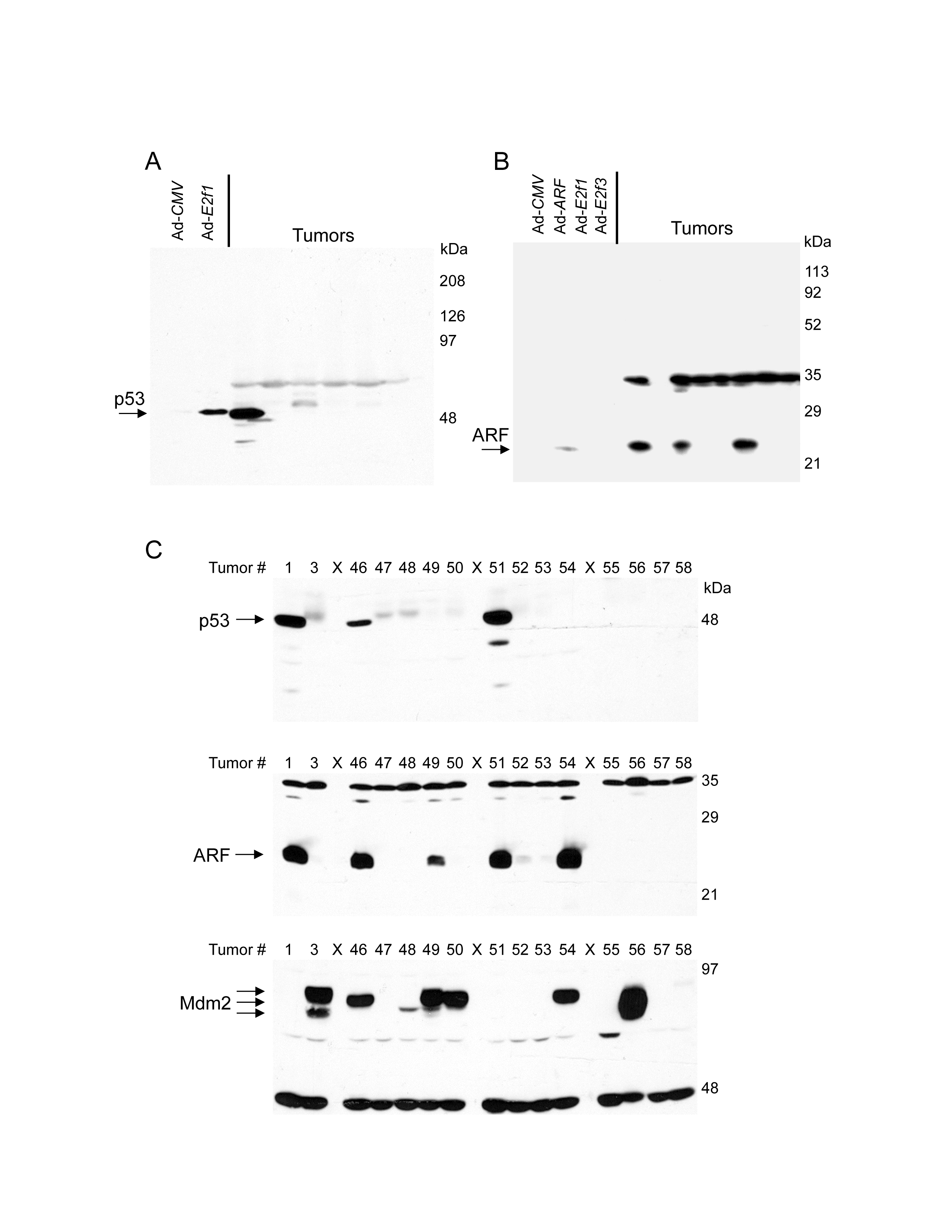

Supplement: Figure S7 — Immunoblot analysis of p53, ARF, and Mdm2 expression in MEFs and Eμ-myc tumors. (A) p53 protein was assessed in MEFs infected with either control adenovirus or adenovirus expressing E2F1, and in several tumor samples. Forced overexpression of E2F1 induces accumulation of p53 [13], and the same species was evident in one of the tumors. The p53 protein overexpressed in some Eμ-myc tumors represents mutant forms that accumulate to very high levels because they fail to induce Mdm2 to trigger their own destruction [8]. (B) ARF protein was assessed in MEFs infected with various recombinant adenoviruses, one of which was Ad-Arf, and, in several tumors, some of which overexpress ARF. (C) p53, ARF, and Mdm2 expression assessed in the same set of Eμ-myc tumors. While no specific controls were used for Mdm2 protein, assorted species of the expected size and complexity were identified using the antibody referenced by Eischen et al. [8]. In common with earlier studies, p53 overexpression was associated with ARF overexpression, ARF overexpression also occurred independently in additional tumors, and Mdm2 overexpression patterns were complex. (1.97 MB TIF) [file pgen.1000640.s007.tif]

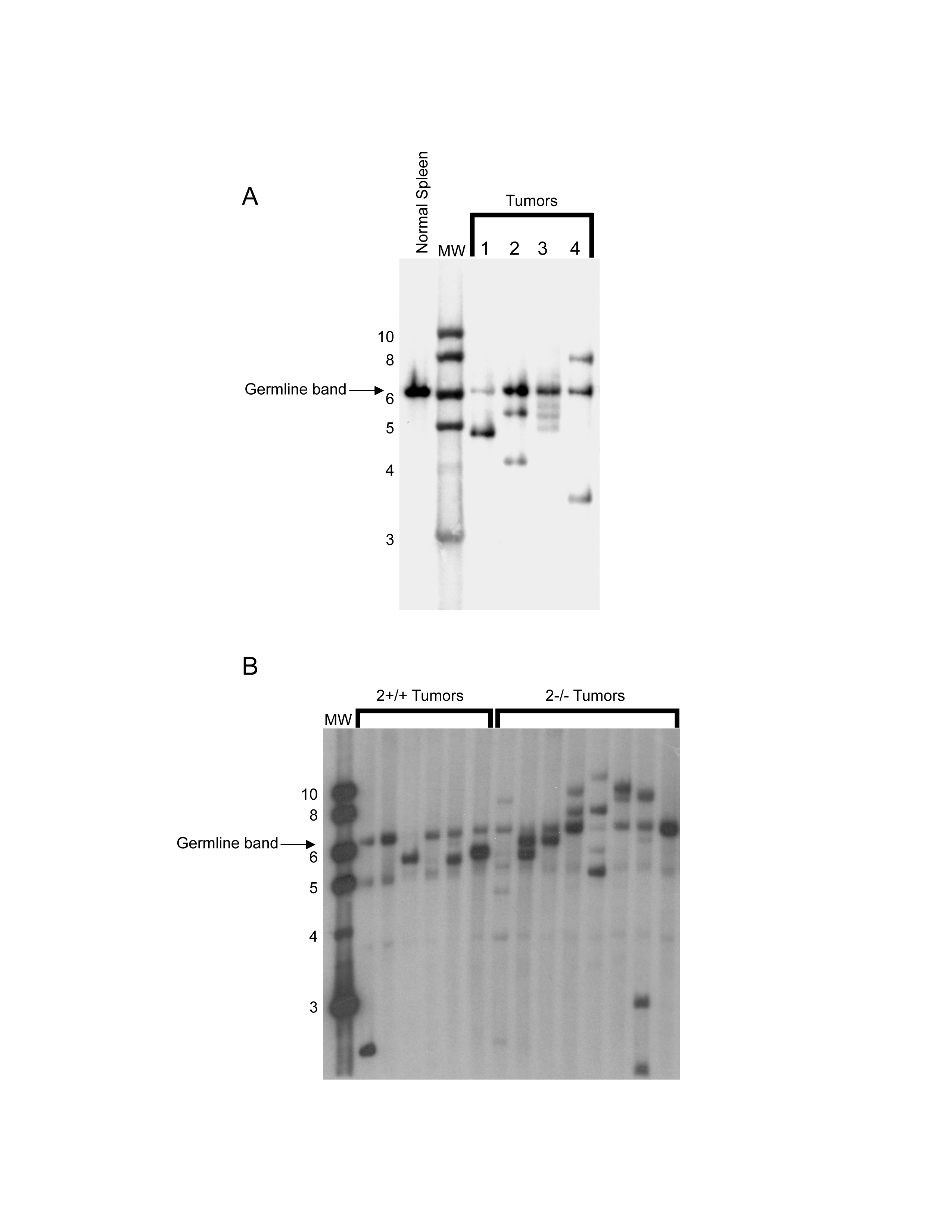

Supplement: Figure S8 — Tumor clonality as determined by Igh locus rearrangement patterns. (A) Normal spleen DNA and tumor DNAs were digested with EcoRI, agarose gel fractionated, and Southern analysis performed using a heavy chain joining region probe. The arrow indicates the 6.5 kb germline fragment in normal spleen DNA, while in tumor samples additional bands were evident, representing rearranged Igh alleles. Most commonly, in addition to the residual germline band, there were two equimolar fragments bearing the heavy chain joining region that resulted from recombination at both Igh alleles (see tumor 2 and tumor 4). The single rearranged fragment seen for tumor 1 probably reflects two co-migrating fragments. In the case of tumor 3, the dominance of the germline band suggests that the sample largely comprised normal cells, and indeed the notes describing the dissection suggested that lymphoma had barely initiated. As described in the figure legend for Figure 5A, lymphomas were characterized as monoclonal if there were zero, one, or two bands in addition to any germline band, as biclonal if there were three or four bands in addition to any germline band, and oligoclonal if there were five or more bands in addition to any germline band. (B) As an additional example of this analysis, the Igh rearrangement patterns for several E2f2+/+ and E2f2 −/− Eμ-myc tumors are displayed. (1.25 MB TIF) [file pgen.1000640.s008.tif]

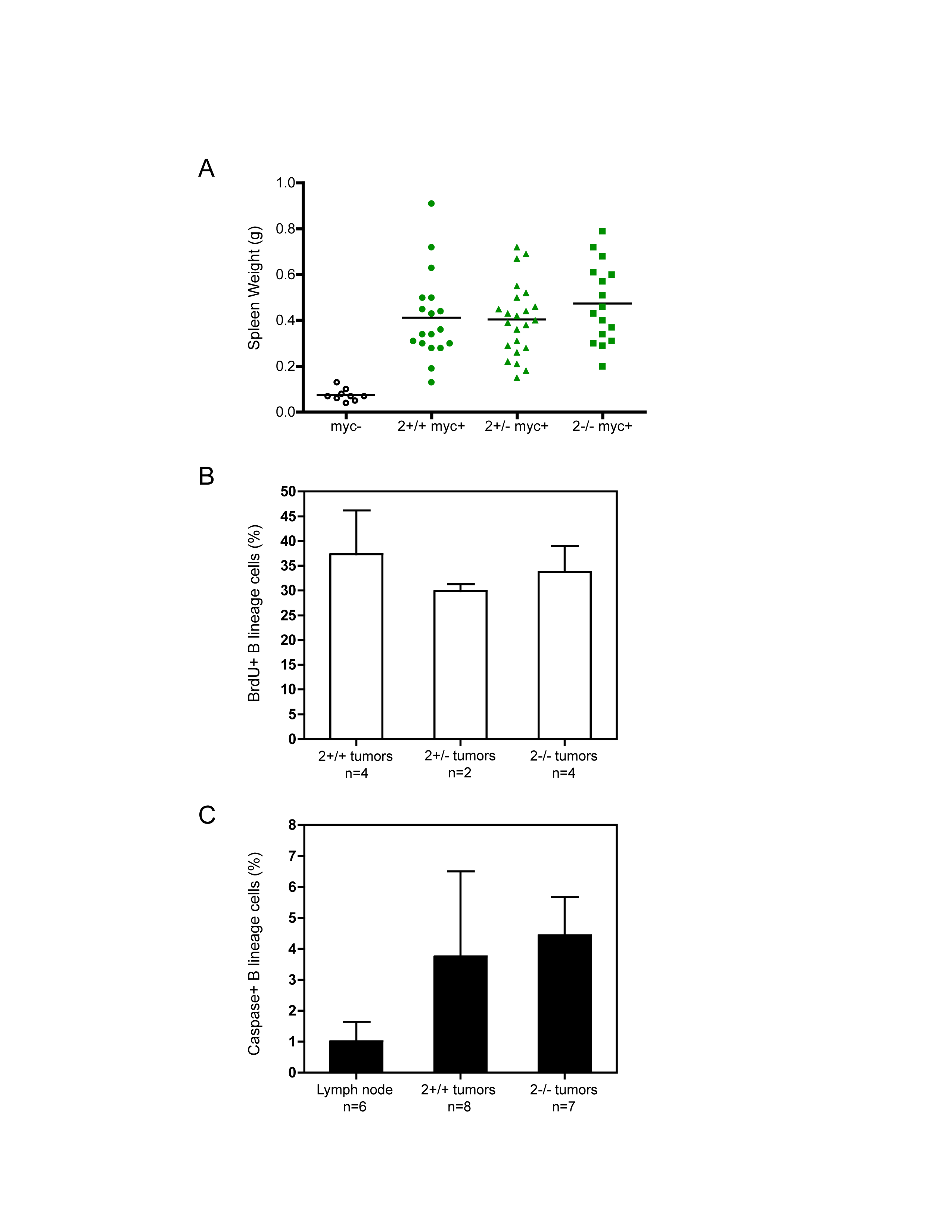

Supplement: Figure S9 — The E2f2−/− mice exhibit standard Eμ-myc lymphoma. (A) Comparison at time of dissection of spleen weights from tumor-bearing E2f2+/+ (n = 18), E2f2+/− (n = 23), and E2f2−/− (n = 16) mice and non-transgenic mice (n = 9). (B) Proliferation of B lineage (B220+) lymphoma cells. Tumorous mice were injected with BrdU and two hours later lymphomas dissected. BrdU incorporation into the DNA of B220+ lymphoma cells was assessed by flow cytometry. (C) Apoptosis of B lineage (B220+) lymphoma cells. Freshly isolated lymphoma cells and normal lymph node cells were isolated and stained with 7-AAD and antibodies to B220 and activated caspase 3. B220+ cells were designated as apoptotic if positive for activated caspase 3 and either negative or positive for 7-AAD. (0.21 MB TIF) [file pgen.1000640.s009.tif]

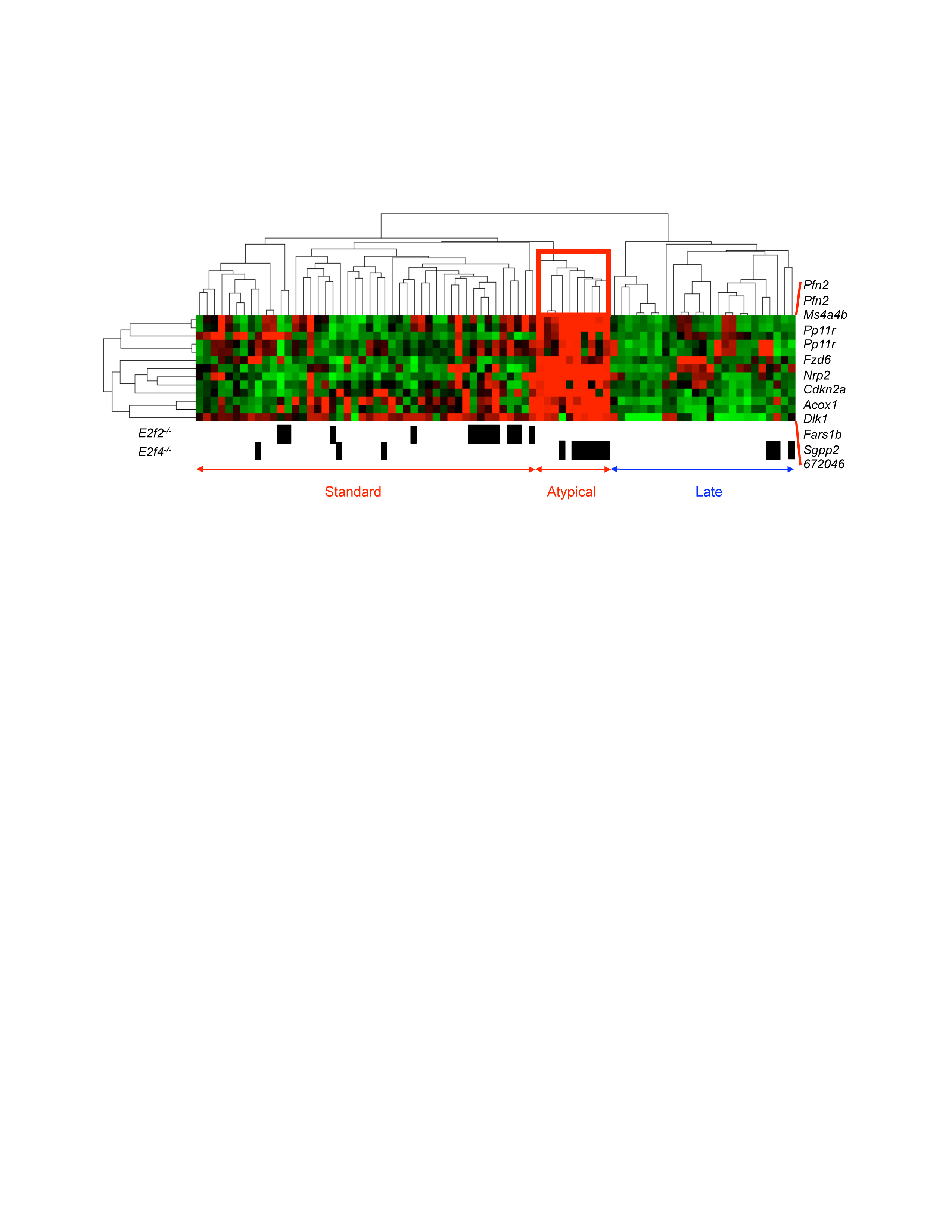

Supplement: Figure S10 — The expression pattern of genes associated with the Expression levels are displayed with genes as rows and samples as columns and shown by a heatmap in which high expression is indicated by red and low expression by green. (0.36 MB TIF) [file pgen.1000640.s010.tif]
