# Supplementary material for: A Role for E2F Activities in Determining the Fate of Myc-Induced Lymphomagenesis
Source: PLoS Genet. 2009 Sep 11;5(9):e1000640. doi: 10.1371/journal.pgen.1000640 (PMC2729385; doi:10.1371/journal.pgen.1000640)
Supplement: Text S1 — Supporting materials and methods. (0.06 MB DOC) [file pgen.1000640.s011.doc]

**Supporting Materials and Methods**

**RT-PCR RNA analysis.** For the survey of E2F expression, selected tissues were dissected from three wild type 3-month-old mice. For the bone marrow, spleen, and thymus tissues, single cell suspensions were made by pressing the tissues between ground glass slides and passing the cells through nylon netting; after gentle centrifugation, erythrocytes were removed by hypotonic lysis and the cells washed in phosphate buffered saline. The mouse tissues were disrupted in guanadinium isothiocyanate using a tissue homogenizer (PowerGen 125, Fisher), and the RNA purified via cesium chloride centrifugation, and quantitated by A260 absorbance. Quantitative RT PCR was performed on each sample in triplicate using the ABI 7600 HT machine and single step RT PCR SYBR green reagents from Qiagen. Analysis was completed with ABI SDS software and relative levels were determined using the delta, delta CT method with GAPDH as an internal control. Melting curve analysis was performed to ensure specific products. Primers used for quantitative RT PCR: mouse *GAPDH* (TCATGACCACAGTGGATGCC and GGAGTTGCTGTTGAAGTCGC); mouse *E2f1* (CGATTCTGACGTGCTGCTCT and CAGCGAGGTACTGATGGTCA); mouse *E2f2* (GCGCATCTATGACATCACCA and CGGGTGGGGTCTTCAAATAG); mouse *E2f3a* forward (CCAGCAGCCTCTACACCAC); mouse *E2f3b* forward (CTTTCGGAAATGCCCTTACA); mouse *E2f3a/3b* common anchor (GGTACTGATGGCCACTCTCG); and mouse *E2f4* (CACTGAGGACGTCCAGAACA and gatgggcacctctagactgg).

**Western analysis.** For the survey of E2F2 and E2F4 protein expression, selected tissues were dissected from wild type and nullizygous 3-month-old mice. Single cell suspensions of spleen, thymus, and liver were made by pressing the tissues between ground glass slides and passing the cells through nylon netting. Erythrocytes were removed from bone marrow, spleen, thymus and liver samples by hypotonic lysis and the cells washed in phosphate buffered saline. The other tissues were washed in PBS and directly frozen. Samples were then weighed, ground to a powder in liquid nitrogen and resuspended in 1 ml per 0.2g weight of 60 mM Tris (pH 6.8)/1% SDS, boiled, sonicated, and any remaining debris removed by centrifugation. For the analysis of p27^Kip1^ protein, B lineage cells were isolated from spleens of weanling mice (SpinSep Mouse B Cell Enrichment Cocktail, Stem Cell Technologies) and extracts prepared as above. Protein was quantitated using the BCA Protein Assay Reagent Kit (Pierce). Samples (100 µg) were boiled in sample buffer and subjected to SDS-PAGE on 8.5% polyacrylamide gels and western analysis. Each E2F protein was detected using a rabbit polyclonal antibody directed against the C-terminus from Santa Cruz Biotechnology [E2F2 sc-633 and E2F4 sc-1082]. p27^Kip1^ was detected using a polyclonal antibody (C-19 sc-528 at 1:1000, Santa Cruz Biotechnology). Equal protein loading was verified by staining blots with Ponceau Red (0.2% ponceau red in 3% trichloroacetic acid). Protein loading was also evaluated by immunoblotting with a monoclonal anti-alpha tubulin antibody (Sigma-Aldrich, 1:5000) and a monoclonal anti-actin antibody (C-2 sc8432 at 1:1000, Santa Cruz Biotechnology). However, levels of actin and tubulin were sensitive to Eµ-*myc* transgene status and tumor progression limiting their usefulness as monitors of protein loading.

##### Proliferation of splenic B cells from normal and pretumorous mice. Fourteen hours before analysis, mice were injected with 100 mg/kg BrdU. Splenic mononuclear cells were collected and stained with B220 antibody to identify B lineage cells, with anti-BrdU antibodies and with 7-AAD. BrdU Flow Kit reagents and directions were followed (BD/Pharmingen).

**Assessment of lymphoma proliferation and apoptosis.** For proliferation assays, mice bearing tumors were injected intraperitoneally with BrdU (100 mg/kg) and dissected 2.5 hours later. Lymphoma cells, originating from an enlarged inguinal node, were stained with B220 antibody, fixed, permeabilized, DNase-treated, stained with anti-BrdU antibody and 7-AAD, and viable cells assessed for BrdU incorporation by flow cytometry. For apoptotic assays, cells originating from the mesenteric lymph node in normal mice and from an enlarged inguinal node in tumorous mice were stained with B220 antibody and 7-AAD, then fixed and permeabilized, stained with antibody to activated caspase-3 and analyzed by flow cytometry. B220+ cells were designated apoptotic if they were positive for activated caspase-3 and negative or positive for 7-AAD.
